# Therapeutic targeting of FOSL1 and RELA-dependent transcriptional mechanisms to suppress pancreatic cancer metastasis

**DOI:** 10.1038/s41419-025-07810-x

**Published:** 2025-07-09

**Authors:** Joana E. Aggrey-Fynn, Meghana Manjunath, Ashish Rajput, Amro M. Abdelrahman, Julia Thiel, Mark J. Truty, Andrew Clark, Meng Dong, Steven A. Johnsen

**Affiliations:** 1 Robert Bosch Center for Tumor Diseases (RBCT), Stuttgart, Germany; 2 Department of Biochemistry and Molecular Biology, Mayo Clinic, Rochester, MN USA; 3 Department of Surgery, Mayo Clinic, Rochester, MN USA; 4 Dr. Margarete Fischer-Bosch Institute for Clinical Pharmacology and University of Tübingen, Stuttgart, Germany; 5 Institute of Cell Biology and Immunology, University of Stuttgart, Stuttgart, Germany; 6 University of Tübingen, Tübingen, Germany

**Keywords:** Pancreatic cancer, Preclinical research

## Abstract

Pancreatic ductal adenocarcinoma (PDAC) is a highly aggressive cancer often diagnosed at an advanced stage, leading to a poor prognosis. The tumor microenvironment (TME) plays a crucial role in driving metastasis, with inflammatory signaling pathways contributing to tumor progression and therapy resistance. However, the combined effects of inflammatory and oncogenic signaling on the epigenetic regulation of PDAC metastasis are poorly understood. Here, we demonstrate that tumor necrosis factor-alpha (TNFα) and epidermal growth factor (EGF) signaling converge to regulate PDAC cell migration through the activation of NF-κB and AP-1 transcription factors. Using single-cell RNA sequencing, in vitro and in vivo models, we show that the simultaneous activation of these pathways with TNFα and EGF cooperatively induces the expression of genes associated with cell motility and migration. Consistently, combinatorial induced genes are co-regulated by the transcription factors FOSL1 and RELA. Remarkably, inhibition of NF-κB transcriptional activity with a glucocorticoid receptor (GR) mixed agonist significantly reduced PDAC cell migration by decreasing RNA polymerase II recruitment to target genes. These findings reveal a novel mechanism by which inflammatory and oncogenic pathways cooperate to drive PDAC metastasis and highlight the therapeutic potential of GR agonists in mitigating tumor cell migration. Our study offers promising avenues for developing mechanism-based therapeutic strategies in PDAC management.

## Introduction

Despite advances in understanding pancreatic ductal adenocarcinoma (PDAC) biology and treatment, it remains one of the most aggressive malignancies, with a 5-year survival rate less than a 10% for all patients and only 1% for advanced cases [1]. The high mortality rate is largely due to its aggressive nature and early metastasis [2], with over half of PDAC patients having metastases at diagnosis and metastatic reoccurrence in about 75% of surgically resected cases [3, 4].

PDAC is associated with various genetic mutations and alterations, with *Kirsten rat sarcoma virus* (*KRAS*) mutations in over 90% of cases [5]. Oncogenic *KRAS* functions by activating the mitogen-activated protein kinase (MAPK) pathway, activating genes linked to cell proliferation, invasion, metastasis, and therapy resistance [6, 7]. A major *KRAS* imbalance favoring the mutant allele is seen in 4% of primary tumors versus 29% of metastatic disease [8]. EGFR signaling is crucial for KRAS-induced precursor lesion formation during acinar-to-ductal metaplasia (ADM) [9–11].

Though mutant KRAS is essential for tumor initiation and maintenance, on its own, it is a weak oncogene in PDAC [5]. Inflammation triggers the rapid transition of acinar cells to ADM, which typically resolves through acinar re-differentiation [12]. However, *KRAS* mutation causes persistent ADM, resulting in pancreatic intraepithelial neoplasia formation [13, 14]. The collaboration between inflammatory signaling and *KRAS* oncogenic activation is attributed to epigenetic modifications in epithelial cells, hastening tumor development. Macrophages secrete pro-inflammatory cytokines, including tumor necrosis factor-alpha (TNFα), which activate downstream Nuclear Factor-kappa-B (NF-κB) signaling and promote ADM and precursor lesion formation [15–17].

PDAC is characterized by a highly reactive inflammatory desmoplastic stroma. The crosstalk between tumor and inflammatory cells stimulates cytokine production, favoring an immunosuppressive phenotype [18, 19]. This is associated with the presence of a subset of immune regulatory cells that fuel inflammation, angiogenesis, and matrix deposition [20–22]. Notably, TNFα-secreting macrophages can drive PDAC cells toward a more aggressive identity [23] whereby tumor cells and macrophages display extensive crosstalk [24]. Overall, the interaction between cancer cells and the TME is highly dynamic and significantly contributes to PDAC progression.

In this study, we elucidated the epigenetic and transcriptional mechanisms underlying the convergence of inflammatory NF-κB signaling with oncogenic MAPK signaling to regulate metastasis in pancreatic cancer. Analysis of PDAC patient samples confirmed the proximity of macrophages to tumor cells expressing the convergent NF-κB/AP1 target gene, *IL1B*. These signaling pathways converge at the genomic level, where activation leads to the co-binding of FOSL1 and RELA at specific genomic regions, promoting cell migration. FOSL1/RELA-mediated epigenetic reprogramming can be countered using glucocorticoid receptor (GR) agonists. Glucocorticoids, known for suppressing NF-κB-dependent activation of inflammatory gene expression, are nearly universally used during PDAC chemotherapy to alleviate side effects. This highlights their potential to prevent further metastasis.

Our findings uncover transcriptional regulatory mechanisms downstream of the major inflammatory and oncogenic signaling pathways, which are important for cell migration in pancreatic cancer. Notably, this work provides a mechanistic rationale to suggest that standard of care supportive therapy with glucocorticoids may have a beneficial effect in suppressing PDAC cell migration during chemotherapy treatment.

## Results

### TNFα-expressing macrophages promote a migratory cell identity in PDAC

**Fig. 1:**
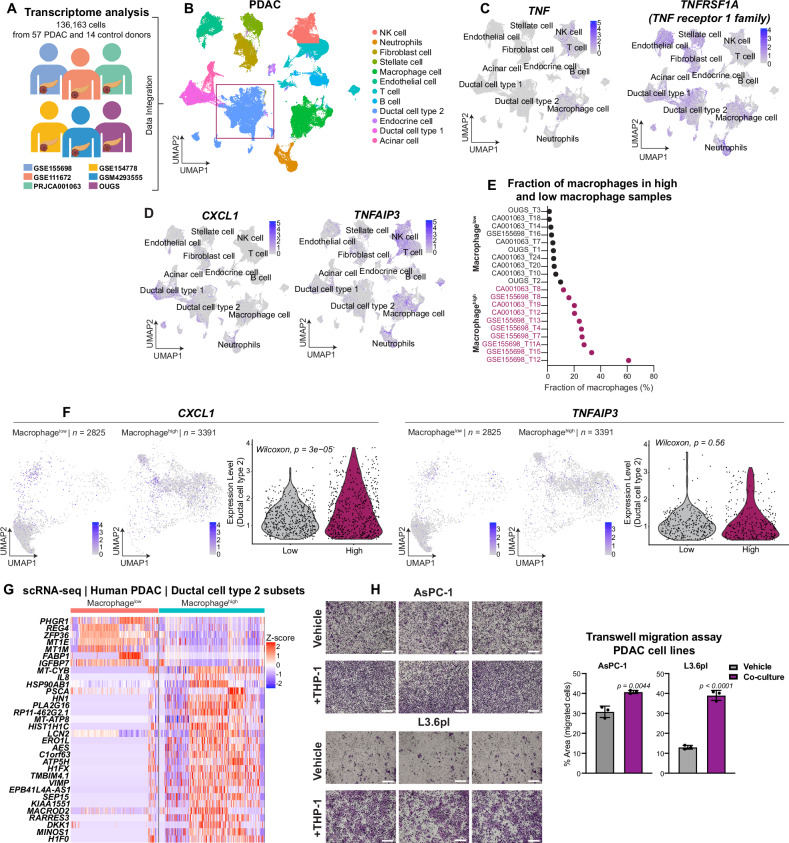
TNFα-producing macrophages in TME enhance the ability to promote cell migration in PDAC. **A**, **B** Schematic of published scRNA-seq data (left) and uniform manifold approximation and projection (UMAP) (right) of six published scRNA-seq data from patients with PDAC (*n* = 136,163 cells from *n* = 71 donors). Each cell is colored by cell subset. **C** UMAPs showing the expression patterns of the *TNF* family gene and the *TNF* receptor *TNFRSF1A* (*n* = 136,163). **D** UMAPs showing cells that express *TNF*-induced NF-κB target genes *CXCL1* and *TNFAIP3* (*n* = 136,163). **E** Percentage fraction of macrophages over total number of cells per donor sample (*n* = 20) representing highest (*n* = 10; purple) and lowest (*n* = 10; gray). **F** UMAPs (left) and violin plots (right) comparison of the ductal cell type 2 cluster of *CXCL1* and *TNFAIP3* expression in the macrophage^high^ and macrophage^low^ groups. **G** Heatmap of scaled expression after differential expression analysis using DESeq of the top up- and downregulated genes in the high vs low macrophage cohorts. Some analysis methods were adapted from Chijimatsu et al. 2022 (reference in supplementary material. **H** Representative crystal violet staining and quantification (mean ± s.d.) of migrated cells (*n* = 3) from the transwell co-culture migration assay of AsPC-1 (top; unpaired *t*-test) and L3.6pl (bottom; unpaired *t*-test) cells with THP-1 cells (*n* = 3). Scale bars, ×4 magnification.

To investigate the relationship between tumor-associated macrophages and tumor cells in PDAC, we analyzed six publicly available single-cell RNA-seq datasets from human PDAC patients (Fig. 1A). As previously described, we identified “ductal cell type 2” as malignant based on poor prognosis markers *KRT19*, *KRT7*, and *CEACAM1/5/6* [25]. *TNF* (gene encoding TNFα) expression was high in macrophages, T cells, and B cells, while TNF receptor 1 family (*TNFRSF1A*) was prominent in all cell types, especially ductal cell type 2 and macrophages (Fig. 1B and C). Given that TNF proteins primarily activate the NF-κB pathway, we confirmed pathway activation by observing the expression of NF-κB target genes *CXCL1* and *TNFAIP3* across different cell types, including malignant ductal cells and macrophages (Fig. 1D). This indicates active NF-κB signaling in various PDAC cell types.

In order to examine macrophage-tumor cell crosstalk in PDAC, we categorized patient tumor samples based on macrophage abundance and *TNF* expression. From this, we selected 10 samples with the highest and lowest macrophage counts (macrophage^high^ and macrophage^low^) and *TNF* expression levels (*TNF*^high^ and *TNF*^low^) (Figs. 1E and S1a). To minimize data integration biases, we separately analyzed a single dataset [26], using similar categorizations (Fig. S1b). The macrophage^high^ ductal cell type 2 samples showed a higher number of tumor cells expressing the NF-κB target genes *TNFAIP3* and *CXCL1* compared to macrophage^low^ samples (Figs. 1F and S1c, d), consistent with the known role of TNF in activating NF-κB signaling in PDAC [23]. Further comparison using established PDAC molecular subtype signatures [8] indicated that macrophage^high^ and *TNF*^high^ groups correlate with an intermediate to basal PDAC phenotype, often linked to poor prognosis and metastasis (Fig. S1e, f).

Macrophage^high^ and *TNF*^high^ samples showed increased expression of genes associated with poor prognosis, inflammation, and metastasis, such as *ERO1L*, *PSCA*, *NEAT1*, *HN1*, *LCN2*, *DKK1*, *IL8*, *RARRES3*, *PLCG2*, and *SNCG* (Figs. 1G and S1g, h; references in Supplementary Material). To confirm the role of macrophage-tumor cell interactions in enhancing cell migration, we performed a co-culture assay with the PDAC cell lines AsPC-1 and L3.6pl, the differentiated monocyte-like THP-1 cells (macrophages). Co-culture significantly increased PDAC cell migration (Fig. 1H), indicating that a macrophage-TNF inflammatory environment promotes pancreatic cancer cell migration.

### EGF and TNFα cooperatively stimulate PDAC cell migration

**Fig. 2:**
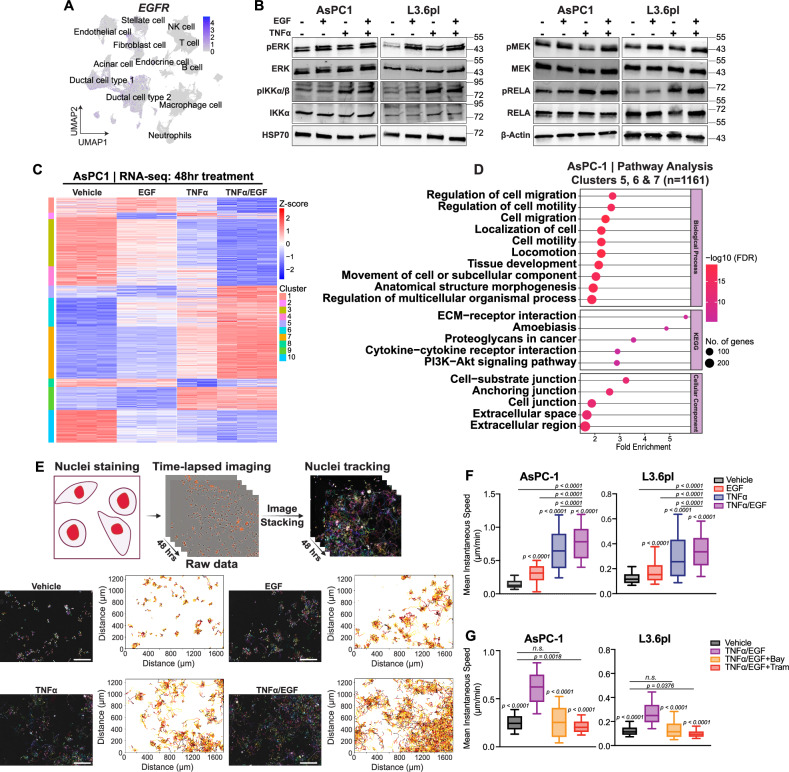
EGF and TNFα cooperatively stimulate cell migration PDAC. **A** UMAP of EGFR expression from the scRNA-seq dataset in Fig. 1a. **B** Western blot for MAPK (pERK, ERK, pMEK, MEK) and NF-κB (pIKKα/β, IKKα, pRELA, RELA) pathway proteins in AsPC-1 and L3.6pl cells following EGF and TNFα treatments for 30 min (right). Representative of *n* = 3 independent experiments. HSP70 and β-Actin serve as loading controls. **C** Heatmap of the differentially expressed genes following RNA-seq on AsPC-1 cells treated with EGF and TNFα for 48 h. Unbiased clustering analysis was done after differential expression with DESeq2. One TNFα-treated replicate (replicate 1) was excluded due to low read depth and outlier behavior in PCA and z-score heatmap analyses, which indicated a sequencing error. **D** Pathway analysis for biological processes, KEGG, and cellular components enriched for the genes in clusters 5, 6, and 7. The top pathways were selected based on FDR values. **E** Single-cell migration tracking migration assay captured every 15 min over 48 h in AsPC-1 cells (magnification = ×10). **F** Mean instantaneous speed calculated from migrated tracks for AsPC-1 and L3.6pl following vehicle, EGF, TNFα, combined (TNFα/EGF) treatments (one-way ANOVA; Dunnett’s multiple comparisons compared to vehicle). **G** Mean instantaneous speed calculated from migrated tracks for AsPC-1 and L3.6pl following vehicle, TNFα/EGF, Bay 117082, and trametinib treatments (one-way ANOVA; Dunnett’s multiple comparisons compared to TNFα/EGF).

The epidermal growth factor (EGF) signals via the EGF receptor (EGFR) and is a key activator of the MAPK signaling pathway in PDAC and other tumors. EGF binding to EGFR triggers receptor tyrosine kinase activity and activates the RAS/RAF/MEK/ERK MAPK cascade [27]. Notably, autocrine EGF signaling further enhances KRAS-driven pancreatic transformation and MAPK signaling [9]. *EGFR* was confirmed to be strongly expressed in the ductal cell type 2 population (Fig. 2A), implicating EGF-induced KRAS/MAPK signaling in PDAC. Thus, we examined the effects of cooperatively activating MAPK and NF-κB signaling by treating AsPC-1 and L3.6pl cells with EGF and TNFα. We examined the kinetics of NF-κB activation in response to TNFα and observed strong activation of downstream genes already after 30 min (Fig. S2a). We also examined whether individual or combined stimulation of downstream signaling influenced one another’s activity and observed that the phosphorylation of downstream targets was similar in the combined treatment compared to individual treatments (Fig. 2B), suggesting that cooperative activity likely occurs through signal integration, rather than direct pathway crosstalk. In order to investigate this, we next performed mRNA-seq analysis to examine the extent of gene expression changes and alterations of cellular identity after single and combined treatments. This approach revealed distinct gene expression patterns, supporting that a specific, disease-relevant gene set was activated by combined EGF and TNFα signaling activation (Figs. 2C and S2b). Pathway analysis showed enrichment in genes associated with inflammatory signaling, cell motility, and migration (Figs. 2D and S2c). Gene set enrichment analysis indicated significant upregulation of metastasis-related genes with EGF and TNFα combined treatment (Fig. S2d). Collectively, these findings suggest that EGF and TNFα signaling converge to activate cell migration in PDAC by transcriptional activation of a specific set of motility-related genes.

To more precisely measure the effects of the convergent signaling on cell motility, we established a single-cell tracking migration assay which revealed significantly increased cell migration in AsPC-1 and L3.6pl after co-treatment with EGF and TNFα (Figs. 2E, F and S2e; Supplementary Videos). This effect was corroborated in additional PDAC cell lines, including Panc1, HPAFII, and BxPC3 (Fig. S2f). Boyden chamber assays confirmed increased migration with combined treatment (Fig. S2g). The direct role of the EGF-mediated MAPK and TNFα-mediated NF-κB pathways was confirmed by treating with Trametinib (MEK1/2 inhibitor) and BAY-117082 (NF-κB inhibitor), which each significantly reduced TNFα/EGF-dependent migration (Fig. 2G). To further strengthen these results, we queried our RNA-seq data for genes upregulated by TNFα/EGF treatments, which are associated with cell migration, including *IL1B*, *IL1A*, *LAMC2*, *MMP1*, *MMP10*, and *IGFBP6,* and verified them independently by qRT-PCR (Fig. S2h). These findings underscore the critical role of convergent MAPK and NF-κB signaling in promoting PDAC cell migration.

### Combined TNFα/EGF co-stimulation induces *IL1B* expression in PDAC cells

**Fig. 3:**
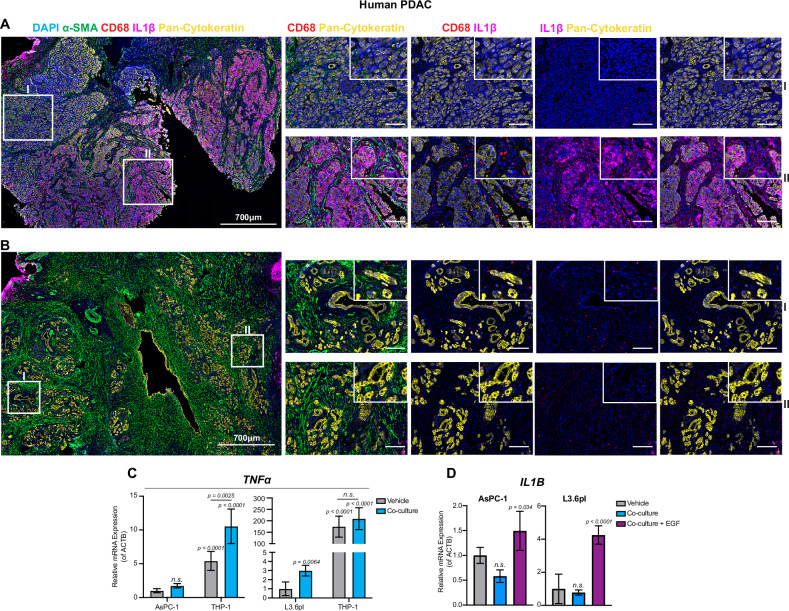
Combined TNFα/EGF co-stimulation induces *IL1B* expression in PDAC cells. **A**, **B** Expression of IL1β (purple), CD68 (red; macrophages), cytokeratin (pan) (yellow; tumor cells), α-SMA (green; fibroblasts), and DAPI (blue; nucleus) detected by multiplex immunofluorescence in PDAC tumor samples (original magnification 700 μm; magnification of white boxes 100 μm; inserts are 40 μm). **C** *TNFα* expression (mean ± s.d.) in AsPC-1 and L3.6pl cells after co-culture with THP-1 cells (*n* = 3) (unpaired *t*-test). **D** *IL1B* expression (mean ± s.d.) in AsPC-1 and L3.6pl cells after co-culture with THP-1 cells (*n* = 3) (unpaired *t*-test).

IL1β is an important mediator of inflammatory signaling in PDAC. The proximity of *IL1B*-positive macrophages to PDAC cells triggers early events leading to transcriptional changes associated with disease progression [24]. However, its role and regulation in tumor cells is less understood. Our RNA-seq analysis identified *IL1B* as a highly upregulated gene in AsPC-1 and L3.6pl cells (log2FC at 6.78 and 8.02, respectively; TNFα/EGF vs Vehicle; Fig. S2h) after TNFα/EGF co-stimulation. Multiplex spectral imaging of patient samples confirmed IL1β expression in PDAC tumors with high macrophage infiltration, indicated by high CD68 (macrophage marker) expression (Fig. 3A, B). These findings support that *IL1B* expression in PDAC cells is related to macrophage infiltration and supports the importance of convergent signaling and cell-to-cell interactions.

To further investigate the relevance of these findings and the interaction between PDAC and macrophages, we examined the effect of co-culturing PDAC tumor cells and macrophage-like cells on the induction of *IL1B* expression. Gene expression analysis confirmed significantly higher TNFα levels in macrophage-like THP1 cells following co-culture with the PDAC cell lines AsPC-1 and L3.6pl (Fig. 3C). Notably, co-culturing PDAC cells with TNFα-producing THP-1 cells resulted in upregulation of *IL1B* (Fig. 3D). These results confirm that the MAPK and TNFα/NF-κB signaling cooperatively induce *IL1B* expression.

### FOSL1 and RELA transcription factors are key for convergent EGF and TNFα signaling

**Fig. 4:**
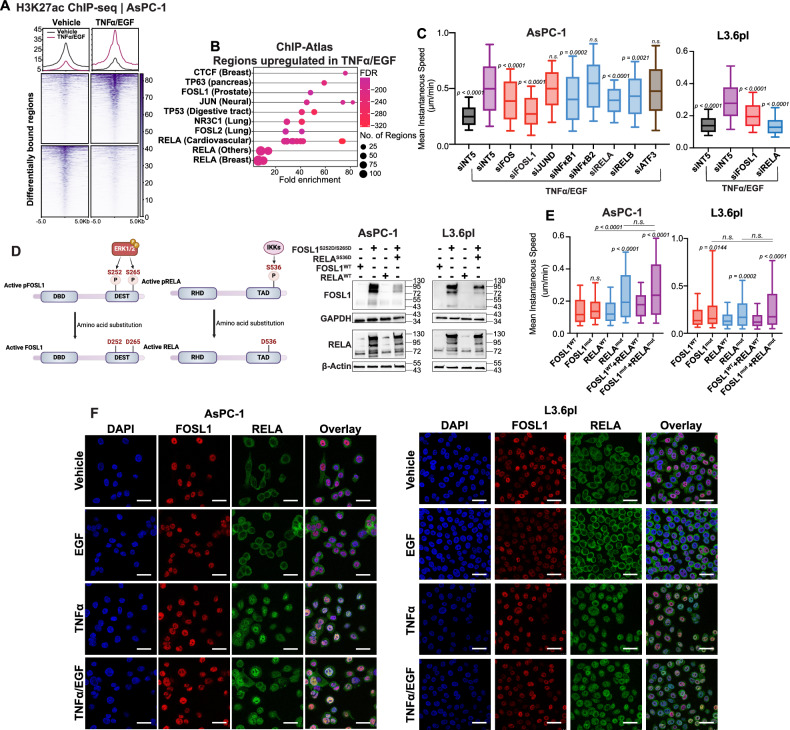
FOSL1 and RELA are the key transcription factors involved in the convergence signaling. **A** ChIP-seq heatmap showing H3K27ac occupancy on genomic regions differentially bound in vehicle and combined treatment in AsPC-1 cells. **B** Dotplot for the top ten transcription factors enriched in H3K27ac upregulated regions in the combined treatment following ChIP-Atlas analysis. The top pathways were selected based on FDR values. Multiple dots indicate multiple datasets for a given factor. **C** Mean instantaneous speed calculated from migrated tracks for AsPC-1 and L3.6pl following knockdown of AP-1 and NF-κB transcription factors (one-way ANOVA; Dunnett’s multiple comparisons compared to siNT5+TNFα/EGF). **D** Schematic models for phosphomimetic mutants of FOSL1 and RELA. Western blot for FOSL1 and RELA in AsPC-1 and L3.6pl following transfections with plasmids containing mCherry and EGFP (for wildtype) and FOSL1^S252D/S265D^ and RELA^S536D^. **E** Mean instantaneous speed calculated from migrated tracks for AsPC-1 and L3.6pl cells transfected with wildtype and mutant FOSL1 and RELA (unpaired *t*-test). **F** Immunofluorescence staining for FOSL1 (red) and RELA (green) showing co-localization following combined treatments in AsPC-1 and L3.6pl (magnification = ×63 magnification with oil immersion).

To identify transcription factors downstream of EGF and TNFα signaling in PDAC, we examined changes in the epigenetic landscape following co-stimulation of both pathways. Differential binding analysis of ChIP-seq data for the active mark H3K27ac, after EGF and TNFα treatment for 30 min, revealed regions with increased H3K27ac marks following the combined treatment. Enrichment analysis showed that these regions were bound by AP-1 factors (FOSL2, FOSL1, JUN) and the NF-κB transcription factor RELA (Figs. 4A, B and S3a, b). To determine which AP-1 and NF-κB transcription factors may be required for the phenotypic effects elicited by combined treatment, we performed single-cell tracking and Boyden chamber migration assays with siRNA-mediated knockdown of AP-1 and NF-κB factors for 24 h. Silencing of either FOSL1 or RELA significantly reduced cell migration (Figs. 4c and S3c). Given the association of FOSL1 with elevated ERK1/2 activity and cancer cell invasion [28, 29], we conducted gain-of-function assays with phosphomimetic mutants of FOSL1 and RELA (Fig. 4d, Supplementary Materials 1 and 2). Mutating serine residues in FOSL1 and RELA to aspartate mimicked active states, and combined expression of FOSL1^S252D/S265D^ and RELA^S536D^ markedly increased cell migration compared to wild-type or individual mutants (Fig. 4E). Immunofluorescence staining confirmed the co-localization of FOSL1 and RELA following combined treatment (Fig. 4f). Additionally, multiplex staining confirmed RELA activation (phosphorylation) in PDAC cells of macrophage-rich, IL1β-positive tumors (Fig. S3g). These data collectively show that co-stimulation with EGF and TNFα promotes PDAC cell migration through the cooperative activity of FOSL1 and RELA, with their knockdown reducing cell migration and phosphomimetic mutants enhancing it.

### FOSL1 and RELA co-occupy specific regions to activate gene transcription

**Fig. 5:**
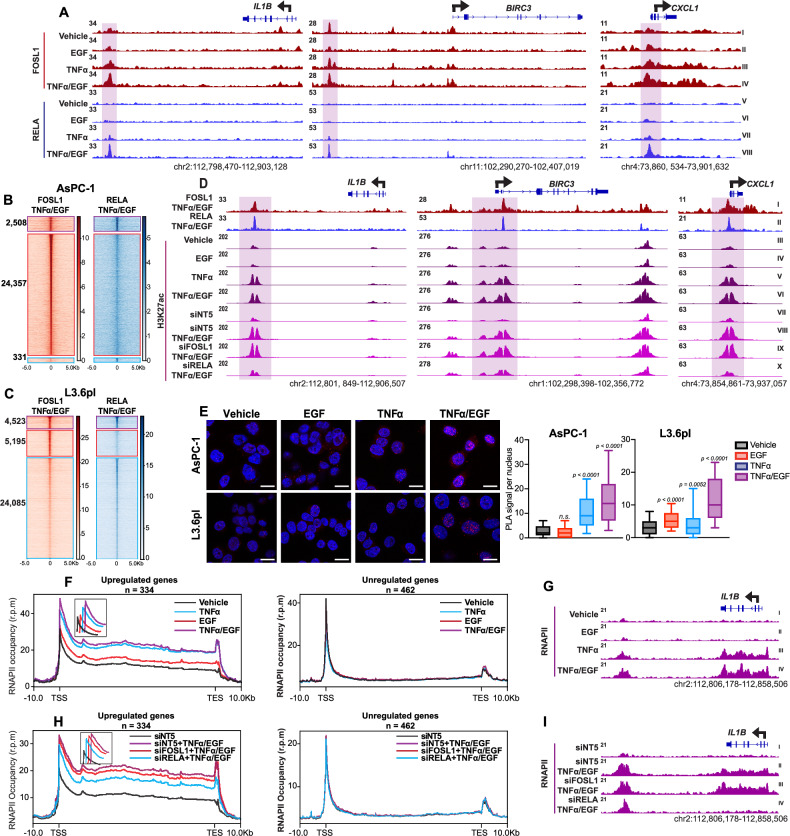
FOSL1 and RELA co-localize to activate gene transcription. **A** Genome browser view (IGV) of AsPC-1 cells at the *IL1B*, *BIRC3*, and *CXCL1* loci showing FOSL1 and RELA ChIP-seq (red and blue, respectively) signals following vehicle, EGF, TNFα, and TNFα/EGF treatments. **B** Heatmaps for FOSL1 and RELA ChIP-seq signals showing FOSL1/RELA dominant (*n* = 2508), FOSL1 dominant (*n* = 24,357), and RELA dominant clusters (*n* = 331). **C** Heatmaps for FOSL1 and RELA ChIP-seq signals showing FOSL1/RELA dominant (*n* = 4523), FOSL1 dominant (*n* = 5195), and RELA dominant clusters (*n* = 24,085) in L3.6pl. **D** IGV tracks showing ChIP-seq signals for FOSL1, RELA, and H3K27ac after EGF, TNFα, and combined treatments with and without siRNA-mediated FOSL1 and RELA knockdowns. **E** PLA showing protein interactions between FOSL1 and RELA in AsPC-1 and L3.6pl (one-way ANOVA; Dunnett’s multiple comparisons compared to vehicle). Each red dot represents a single interaction, and DNA was stained with DAPI (magnification = ×63 magnification with oil immersion). Representative of *n* = 3 independent experiments; boxplots represent the number of PLA signals per 100 cells. **F** Metagene plots showing the binding profiles of RNAPII following vehicle, EGF, TNFα, and TNFα/EGF at the TSS, gene body, and TES of genes upregulated in TNFα/EGF (*n* = 334) and unregulated genes (*n* = 462). **G** IGV tracks of AsPC-1 cells at the *IL1B* gene showing RNAPII ChIP-seq signals following vehicle, EGF, TNFα, and TNFα/EGF treatments. **H** Metagene plots showing the binding profiles of RNAPII following FOSL1 and RELA knockdown at the TSS, gene body, and TES of genes upregulated in TNFα/EGF (*n* = 334) and unregulated genes (*n* = 462). **I** IGV tracks of AsPC-1 cells at the *IL1B* showing RNAPII ChIP-seq signals following FOSL1 and RELA knockdown.

We next examined the genome-wide binding dynamics of FOSL1 and RELA using ChIP-seq after EGF, TNFα, or combined treatments. Global analysis indicated that RELA binding increased with TNFα and combined treatments (Fig. S4a). Consistent with its constitutive nuclear localization and regulation by phosphorylation, FOSL1 displayed significant binding independent of EGF and TNFα treatments (Fig. S4b), although a cluster of FOSL1-bound regions revealed increased H3K27ac occupancy compared to the vehicle (Fig. S4c). Notably, FOSL1 and RELA displayed cooperative binding at enhancer regions near several genes upregulated after 30 min EGF and TNFα co-treatment (*IL1B*, *BIRC3*, and *CXCL1*) (Figs. 5A and S4d). These findings support a crucial role of FOSL1 in the epigenetic regulation of genes cooperatively induced by EGF and TNFα.

Analysis of the binding patterns of FOSL1 and RELA (Figs. 5A and S4d) suggested that the two factors occupy overlapping genomic sites with the combination treatment showing the highest co-occupancy of RELA and FOSL1. To further investigate this, we intersected FOSL1 and RELA peaks and identified a subset of regions co-occupied by both (Fig. 5B, C). This analysis revealed three distinct clusters: FOSL1/RELA-co-occupied, RELA-dominant, and FOSL1-dominant regions across AsPC-1 and L3.6pl cell lines (Fig. 5B, C). Motif analysis showed enrichment of AP-1 and NFκB/Rel family motifs in FOSL1- and RELA-dominant regions, respectively, while co-bound regions displayed motifs for both factors (Fig. S4e). The top enriched RELA motif depicted binding of FOSL1 and NFκB-p50 (RELA-p50 heterodimers). Furthermore, pathway analysis of the two nearest genes in the FOSL1/RELA co-bound regions (as identified by GREAT analysis) revealed enrichment in pathways related to cell migration, motility, and locomotion. This enrichment was observed in comparison to regions that were predominantly bound by either FOSL1 or RELA alone (Fig. S4f–h). These findings suggest that EGF and TNFα signaling cooperatively promote the co-occupancy of FOSL1 and RELA at a select set of enhancer regions to activate a subset of genes (Fig. 5D). We confirmed the physical interaction between FOSL1 and RELA following EGF and TNFα treatments using a proximity ligation assay (PLA) (Fig. 5E). Altogether, the analysis of FOSL1 and RELA binding patterns revealed significant co-occupancy at overlapping genomic sites, which are linked to genes involved in cell migration and motility, emphasizing the cooperative role of EGF and TNFα signaling in gene regulation.

### RELA is essential for epigenetic regulation downstream of EGF and TNFα signaling

To understand the roles of FOSL1 and RELA in the epigenetic regulation of EGF and TNFα signaling, we investigated their binding and H3K27ac occupancy following siRNA-mediated knockdown. Silencing RELA reduced FOSL1 and H3K27ac occupancy, whereas silencing FOSL1 did not appreciably affect either RELA or H3K27ac occupancy (Figs. 5C and S4i). Together, these findings suggest that RELA plays a dominant role in both the epigenetic marking and recruitment of FOSL1 at these regions.

We next sought to further examine the transcriptional mechanism by which FOSL1 and RELA cooperatively activate gene transcription. We therefore examined RNA Polymerase II (RNAPII) occupancy by ChIP-seq after EGF and TNFα treatments and siRNA knockdowns of either FOSL1 or RELA. Both treatments induced RNAPII recruitment and elongation on TNFα/EGF-regulated genes (genes were identified from RNA-seq data after 30-min treatments), with TNFα showing a stronger effect (Fig. 5F, G). Combined EGF and TNFα treatment significantly increased RNAPII recruitment both at transcription start sites (TSS) and across the gene body, with TNFα treatment showing the strongest effect. Consistently, depletion of RELA substantially reduced RNAPII across the entire gene (Fig. 5H, I), confirming RELA as the key factor in driving the cooperative transcriptional program.

### Glucocorticoid receptor (GR) agonists attenuate EGF and TNFα-mediated gene transcription

**Fig. 6:**
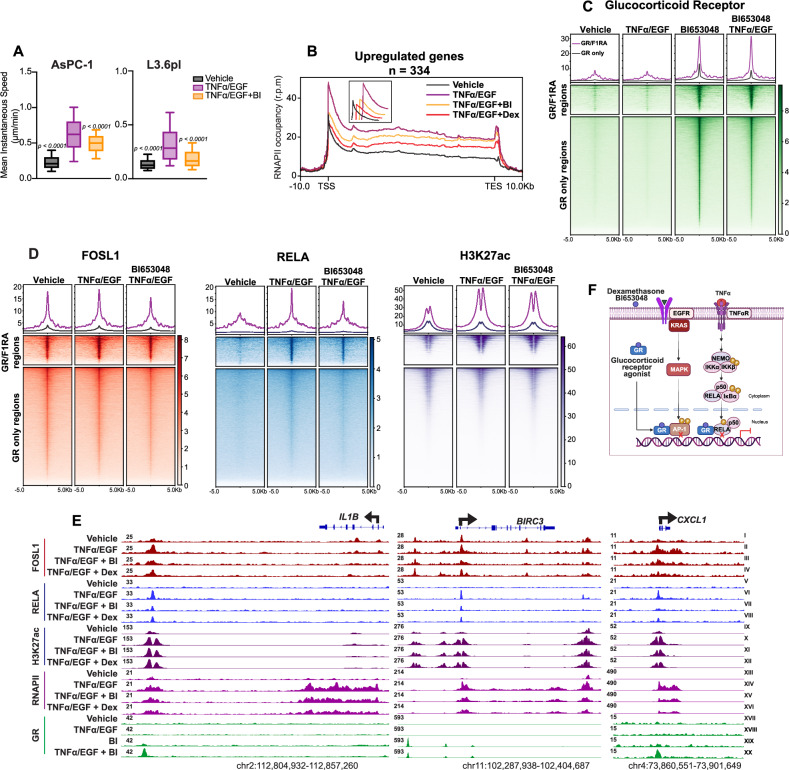
Treatment with glucocorticoid agonists inhibits FOSL1 and RELA binding activity. **A** Mean instantaneous speed calculated from migrated tracks for AsPC-1 and L3.6pl following Dexamethasone and BI 653048 treatments. **B** Metagene plots showing the binding profiles of RNAPII following Dexamethasone and BI 653048 treatments of genes upregulated in TNFα/EGF (*n* = 334). Heatmaps showing GR (**C**), FOSL1, RELA, and H3K27ac (**D**) signals at genomic regions co-occupied by GR, FOSL1, and RELA (GR/F1RA; *n* = 2413) and at GR-only regions genome-wide (*n* = 40,795). **E** IGV showing FOSL1, RELA, H3K27ac, RNAPII, and GR signals at *IL1B*, *BIRC3*, and *CXCL1* following Dexamethasone and BI 653048 treatments. **F** Scheme depicting the mechanism of action of the glucocorticoids Dexamethasone and BI 653048.

We explored the therapeutic potential of targeting FOSL1/RELA-mediated epigenetic regulation and found, through the ChIP-Atlas data in Figs. 4B and S3b, that the transcription factor NR3C1 (encoding the GR) was enriched in both AsPC-1 and L3.6pl cell lines. Based on this, we utilized GR agonists, commonly prescribed to PDAC patients to alleviate chemotherapy side effects [30], as a potential intervention strategy. Glucocorticoids function as anti-inflammatory agents largely by suppressing NF-κB-dependent gene expression [31]. Upon activation, GR translocates to the nucleus and modulates gene transcription through transactivation or transrepression mechanisms, with the transrepression mechanisms mediating the majority of anti-inflammatory effects [28, 29]. To test whether activation of GR signaling may also impair the effects of combined TNFα/EGF signaling pathway activation, we treated cells with the synthetic glucocorticoid dexamethasone (Dex) [28, 29, 32] and the nonsteroidal glucocorticoid receptor mixed agonist BI 653048 (BI) [33]. Both BI and Dex significantly reduced the TNFα/EGF-induced expression of *IL1B* and *MMP1,* as well as impaired TNFα/EGF-driven cell migration (Figs. 6A and S5a). Consistent with GR pathway activation, RNAPII occupancy increased at canonical GR target genes *TSC22D3* and *FKBP5* (Fig. S5b). Additionally, they significantly decreased RELA occupancy at FOSL1/RELA dominant regions, while moderately decreasing FOSL1 with no substantial change in H3K27ac levels (Fig. S5c–e). BI and Dex also decreased RNAPII occupancy across the entire body of TNFα/EGF-induced genes (Fig. 6B).

To further dissect the interaction between GR signaling and convergent TNFα/EGF transcriptional regulation, we performed GR ChIP-seq following treatment with TNFα/EGF, BI, or their combination. GR was enriched at regions co-occupied by FOSL1 and RELA, particularly upon BI treatment (Fig. 6C). In contrast, GR-only binding sites showed little to no signal for FOSL1, RELA, or H3K27ac (Fig. 6D), suggesting that GR is not actively engaged at these sites in this context. These findings indicate that GR is selectively recruited to transcriptionally active, FOSL1/RELA-bound regions and is largely excluded from regions lacking transcriptional activity under these treatment conditions.

These findings demonstrate that GR agonists attenuate TNFα/EGF-driven transcription by selectively interfering with FOSL1/RELA-bound enhancers through a context-dependent transrepression mechanism. Moreover, GR is redirected to these active regulatory elements in the presence of convergent signaling, suggesting a bidirectional regulatory relationship that may constrain its broader anti-inflammatory activity in PDAC.

### Activation of GR transrepression limits in vivo PDAC metastasis

To explore the clinical relevance of our findings, we utilized a translational model with nude mice orthotopically implanted with luciferase-expressing L3.6pl cells, which are characterized by their high metastatic potential to the liver [34]. Nude mice, despite lacking an adaptive immune response, possess macrophages and neutrophils that promote innate immunity, creating a TME rich in macrophages. These macrophages secrete TNFα, facilitating the convergence of EGF and TNFα signaling pathways necessary for activating cell migration and metastasis in an in vivo context.

**Fig. 7:**
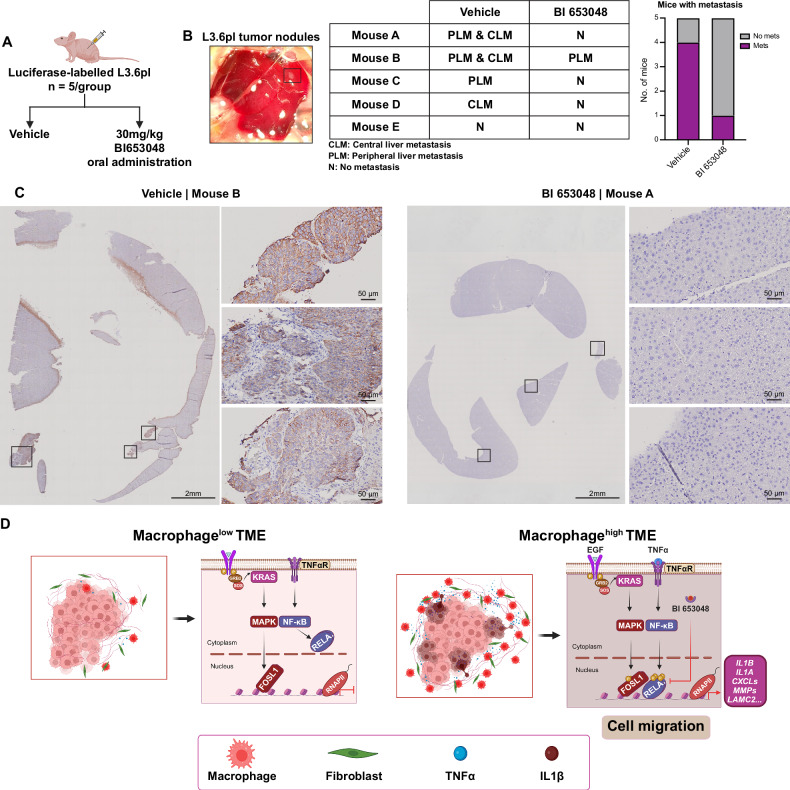
Targeting FOSL1/RELA binding reverses cell migration in vivo. **A** Nude mice were orthotopically implanted with luciferase-expressing L3.6pl cells. Tumor-bearing mice were treated with vehicle and BI 653048 (30 mg/kg; once weekly for 4 weeks) (*n* = 5/group). **B** Summary of the liver examination after harvest at the end of week four. **C** Immunohistochemistry for pan-cytokeratin in tumor sections from harvested liver samples. The sections represent 10% of the total liver size. Samples from vehicle-treated mice exhibit multiple micrometastases, which are absent in *n* = 4 BI 653048-treated mice. Magnification 2 mm; magnification of boxes 50 μm. **D** The graphical scheme describes how macrophages within the TME supply the TNFα needed to activate the EGF and TNFα convergence signaling pathway, driving cell migration in PDAC. This combined signaling leads to the co-binding of FOSL1 and RELA at specific genomic regions, which regulates the recruitment and release of RNAPII, promoting the transcription of migration-related genes. Targeting FOSL1 and RELA binding can inhibit this pathway, reducing the migratory behavior of PDAC cells.

We conducted a two-arm treatment study, administering either vehicle or BI 653048 (BI) to the mice (Fig. 7A, B). BI was selected over Dex due to its enhanced ability to induce transrepression without the dose-limiting side effects or glucocorticoid resistance often associated with Dex [33]. We assessed the impact of BI treatment on metastasis to the liver. Immunohistochemical (IHC) staining for pan-cytokeratin revealed that four out of five vehicle-treated mice developed metastases in the liver (Figs. 7C and S6). In contrast, only one BI-treated mouse showed signs of metastasis (Fig. S6). These findings suggest that targeting the FOSL1/RELA axis with glucocorticoids could offer a strategy for reducing metastasis in PDAC.

In summary, our study reveals details about how inflammatory TNFα and oncogenic EGF/MAPK signaling cooperate to drive cell migration and metastasis in *KRAS*-mutant PDAC. We show that key genes involved in cell migration are activated through the cooperative action of FOSL1 and RELA (Fig. 7C). This signaling pathway is dependent on the simultaneous binding of FOSL1 and RELA to specific genomic regions and targeting this cooperative activity through GR activation could serve as a viable approach to prevent or reduce cell migration in PDAC.

## Discussion

PDAC progresses early to metastatic disease [35], driven by complex transcriptional and epigenetic mechanisms. Targeting these mechanisms could potentially modulate PDAC cell migration, improving treatment outcomes and reducing recurrence [36]. Our study identifies AP-1/FOSL1 and NF-κB/RELA as key transcriptional regulators that cooperatively activate gene expression to promote PDAC cell migration. This aligns with prior research on KRAS-induced FOSL1 and NF-κB signaling in tumor dedifferentiation and metastasis [11, 12, 37–41]. We show inflammation-triggered epigenetic changes via RELA-mediated cooperative FOSL1 binding, activating the H3K27ac histone mark and initiating transcription through RNAPII. Disrupting FOSL1 and RELA interaction with a mixed GR agonist inhibits PDAC cell migration in vitro and in vivo.

Consistent with previous studies by Caronni et al. [24], and Tu et al. [23], our scRNA-seq and multispectral staining support a signaling crosstalk between TNFα-secreting macrophages and tumor cells whereby TNFα-mediated epigenetic reprogramming promotes cell migration and IL1β expression in tumor cells. These findings reveal important insights about the interplay between MAPK signaling, inflammation, and epigenetic reprogramming in PDAC progression and reveal potential therapeutic targets. We further elucidated important insight into transcriptional regulatory mechanisms downstream of TNFα and EGF signaling in PDAC biology through ChIP-seq and functional studies. EGF and TNFα signaling trigger FOSL1 functional activation and RELA binding to genomic regions, respectively, thereby cooperatively upregulating migration-associated genes. Our data suggest that FOSL1 and RELA binding, along with RNAPII recruitment, is predominantly mediated by RELA. TNFα signaling further induces FOSL1 binding specifically at RELA-activated regions, highlighting the cooperative activity of these factors.

Advanced pancreatic cancer remains challenging due to its pathogenesis and resistance to treatments. Emerging therapeutic strategies targeting PDAC molecular drivers, such as *KRAS* mutations, are under intensive investigation. For instance, trials on Sotorasib [NCT05251038] and cancer vaccines are ongoing [42, 43]. Therapies modulating the immune microenvironment, like poly(ADP-ribose) polymerase (PARP) inhibitors combined with immune checkpoint inhibitors, are also being investigated [NCT04753879, NCT02498613, NCT04409002, among others]. Our study reveals that targeting FOSL1 and RELA binding as a novel therapeutic approach, potentially synergizing with existing therapies to improve treatment efficacy.

Given the frequent use of glucocorticoids to alleviate the side effects of chemotherapy, our results have direct translational relevance. Dex, commonly used with chemotherapy, shows pro-apoptotic effects but raises concerns about upregulating pro-survival factors through the transactivation mechanism [30, 44–47]. Mechanistically, our GR ChIP-seq data reveal that GR is preferentially recruited to active, FOSL1/RELA-bound regulatory regions, suggesting a selective transrepression mechanism that may explain its context-dependent effects in PDAC. Using BI 653048, which favors the anti-inflammatory transrepression pathway, may mitigate these risks [33, 48], suggesting BI 653048 may offer a safer option for combination therapy in advanced PDAC, potentially offering new strategies for preventing PDAC metastasis [49].

In summary, our study underscores the cooperative interaction between AP-1/FOSL1 and NF-κB/RELA in regulating migration-specific gene expression. We highlight the pivotal role of macrophage-tumor crosstalk in maintaining this gene expression. These findings suggest that clinical candidates targeting these pathways may effectively inhibit PDAC cell migration, presenting potential new therapeutic strategies for treating this aggressive malignancy.

## Materials and methods

All details are in the Supplementary Materials and Methods.

### Statistics and reproducibility

All images presented are representative of at least three biological replicates, except for single-cell migration assays, where graphs represent ~3000 cells. Statistical significance was assessed using the unpaired *t*-test and ANOVA test with Dunnett’s multiple comparisons. All graphs and statistical analyses were conducted using GraphPad Prism 10.3.1.

## Supplementary information

Supplementary Materials and Methods

Supplementary Figure S1

Supplementary Figure S2

Supplementary Figure S3

Supplementary Figure S4

Supplementary Figure S5

Supplementary Figure S6

AsPC1 cells treated with vehicle (control)

AsPC1 cells treated with EGF

AsPC1 cells treated with TNFa

AsPC1 cells treated with EGF and TNFa

L3.6pl cells treated with vehicle (control)

L3.6pl cells treated with EGF

L3.6pl cells treated with TNFa

L3.6pl cells treated with TNFa and EGF

BxPC3 cells treated with vehicle (control)

BxPC3 cells treated with TNFa and EGF

HPAFII cells treated with vehicle (control)

HPAFII cells treated with TNFa and EGF

Panc1 cells treated with vehicle (control)

Panc1 cells treated with TNFa and EGF

Uncut Western Blot Images

Uncut Western Blot Images

## Data Availability

All raw and processed data are available under the GEO accession numbers GSE278111 and GSE278112.

## Code availability

Custom software was used to perform automated cell tracking and trajectory analysis and is freely available at: https://github.com/Clark-Lab-Stuttgart/incucyte_cell_tracking.
