# Supplementary material for: Therapeutic targeting of FOSL1 and RELA-dependent transcriptional mechanisms to suppress pancreatic cancer metastasis
Source: Cell Death Dis. 2025 Jul 9;16(1):504. doi: 10.1038/s41419-025-07810-x (PMC12241458; doi:10.1038/s41419-025-07810-x)
Supplement: Supplementary file 1 — Supplementary Materials and Methods [file 41419_2025_7810_MOESM1_ESM.docx]

Supplementary Material

Aggrey-Fynn et al.

**Therapeutic Targeting of FOSL1 and RELA-Dependent Transcriptional Mechanisms to Suppress Pancreatic Cancer Metastasis**

Supplementary Figure Legends

Supplementary Materials and Methods

Table S1, siRNAs used in this study

Table S2, Antibodies used in this study

Table S3, Primers used in this study

Table S4, References for genes identified in figures 1h, S1c, and S1f

Supplementary References

Supplementary material 1: Western blots used in the study (AsPC-1)

Supplementary material 2: Western blots used in the study (L3.6pl)

**Supplementary figure legends**

Supplementary figure 1

**TNFα-producing macrophages in TME enhance the ability to promote cell migration in PDAC. a**, Violin plots showing the *TNF* expression levels of *TNF*^high^ (*n* = 10) and *TNF*^low^ (*n* = 10) donor samples. **b**, Projection of cell annotations from the Steele_2020 dataset [1] and of ductal cell type 2 cells in the macrophage^high^ (*n* = 5) and macrophage^low^ (*n* = 5) donor groups. **c and d**, UMAPs (left) and violin plots (right) comparison of the ductal cell type 2 cluster of *CXCL1* and *TNFAIP3* expression in the macrophage^high^ (purple) and macrophage^low^ (gray) groups. **e** **and f**, Pie chart (right) and wishbone plot (left) classifying ductal type 2 cells based on the expression of classical-, intermediate-, and basal-specific genes in the macrophage^high^ and macrophage^low^ (**e**) and *TNF*^high^ and *TNF*^low^ (**f**) groups. The pie chart depicts the number of cells expressing the different molecular subtypes in PDAC. **g**, Heatmap of expression levels after differential expression analysis using DESeq of the top up- and downregulated genes in the *TNF*^high^ and *TNF*^low^. The heatmap depicts genes upregulated in 70% of the cells in the high macrophage cohorts. **h**, Heatmap of expression levels after differential expression analysis using DESeq of the top up- and downregulated genes macrophage^high^ and macrophage^low^ groups. Some analysis methods were adapted from Chijimatsu *et al.,* 2022 [2].

Supplementary figure 2

**EGF and TNFα cooperatively stimulate cell migration PDAC. a**, Quantitative PCR showing significant increase in *CXCL8* and *TNFAIP3* expression following TNFα treatment for 30 minutes (*n* = 3). **b**, Heatmap of the differentially expressed genes following RNA-seq on L3.6pl cells treated with EGF and TNFα for 48 hours. Unbiased clustering analysis was done after differential expression with DESeq2. One TNFα-treated replicate (replicate 1) was excluded due to low read depth and outlier behavior in PCA and z-score heatmap analyses, which indicated a sequencing error. **c**, Pathway analysis for biological processes, KEGG, and cellular components enriched for the genes in clusters 5 and 6. The top pathways were selected based on FDR values. **d**, GSEA analyses on genes upregulated following TNFα/EGF compared to vehicle in AsPC-1 and L3.6pl. the featured gene sets were specially selected for migratory signatures **e**, Generated migration tracks for individual cells of L3.6pl (magnification = 10x). **f**, Generated migration tracks and mean instantaneous speed for BxPC-3, HAPFII, and Panc1 following vehicle and TNFα/EGF treatments (magnification = 10x) (one-way ANOVA). **g**, Crystal violet staining for transwell migration assay for AsPC-1 and L3.6pl following treatments with Vehicle, EGF, TNFα, and TNFα/EGF showing significant increases in migrated cells after TNFα/EGF. **h**, Quantitative PCR showing significant increases of expression of upregulated genes in both AsPC-1 and L3.6pl following TNFα/EGF treatment (*n* = 3) (one-way ANOVA; Dunnett’s multiple comparisons compared to vehicle).

Supplementary Figure 3

**FOSL1 and RELA are the key transcription factors involved in the convergence signaling. a**, ChIP-seq heatmap showing H3K27ac occupancies after DiffBind analysis on Vehicle and combined treatment in L3.6pl **b**, Dotplot for the top ten transcription factors (from ChIP-Atlas) enriched in H3K27ac upregulated regions in the combined treatment. The top pathways were selected based on FDR values. **c**, Trans-well migration assay for AsPC-1 and L3.6pl cells transfected with wildtype and mutant FOSL1 and RELA. **d**, Western blot analysis of FOSL1 and RELA following siRNA-mediated knockdown using four individual siRNA sequences and a pooled mix of all four. The knockdowns presented in main Figure 4 were performed using the pooled siRNA sequences. **e** and **f**, Mean instantaneous speed calculated from migrated tracks for AsPC-1 and L3.6pl following siRNA-mediated knockdown using four individual siRNA sequences and a pooled mix of all four. (one-way ANOVA; Dunnett’s multiple comparisons compared to siNT5 + TNFα/EGF). **g**, Expression of phospho-RELA in IL1β-positive human PDAC sample (original magnification 700μm; magnification of white boxes 100μm).

Supplemental figure 4

**FOSL1 and RELA are the key transcription factors involved in the convergence mechanism. a & b**, Heatmaps of global RELA (**a**) and FOSL1 (**b**) peaks. The sum of all peaks were obtained after concatenating and merging bound regions after vehicle, EGF, TNFα, and TNFα/EGF treatments. **c**, Clustered (Kmeans) heatmap of H3K27ac signals after vehicle, EGF, TNFα, and TNFα/EGF treatments on FOSL1 regions in **b. d**, IGV tracks showing ChIP-seq signals for FOSL1, RELA, and H3K27ac after EGF, TNFα, and combined treatments in L3.6pl cells. **e**, Top enriched motifs at FOSL1/RELA dominant regions defined by HOMER. **f**, Pathway analysis for biological processes for FOSL1/RELA dominant, FOSL1 (**g**), RELA (**h**), regions showing the top 15 enriched pathways. **i**, ChIP-qPCRs for FOSL1, RELA, and H3K27ac on FOSL1 and RELA co-occupied regions at the *IL1B* loci (*n* = 3; purple box) (one-way ANOVA; Dunnett’s multiple comparisons compared to siNT5+TNFα/EGF).

Supplementary Figure 5

**Treatment with glucocorticoid agonists inhibits FOSL1 and RELA binding activity. a**, Quantitative RT-PCR showing significant decrease in *IL1B* and *MMP1* expression following Dexamethasone and BI 653048 treatments (*n* = 3) (one-way ANOVA; Dunnett’s multiple comparisons compared to TNFα/EGF). AsPC-1 cells were pre-treated with Dexamethasone (30nM) and BI 653048 (300nM) for 15 minutes followed by combination treatment for 30 minutes. **b**, IGV of RNAPII signals at *TSCCD3* and *FKBP5* following Dexamethasone and BI 653048 treatments. **c**, Heatmaps for FOSL1, RELA (**d**), and H3K27ac (**e**) ChIP-seq signals on FOSL1/RELA co-occupied regions (*n* = 2,508).

Supplementary Figure 6

**Targeting FOSL1/RELA binding reverses cell migration *in vivo*.** Immunohistochemistry for pan-cytokeratin in tumor sections from the reminder four harvested liver samples. The sections represent 10% of the total liver size. Magnification 2mm; magnification of boxes 50μm.

**Supplementary Materials and Methods**

**Cell Culture**

AsPC-1 (RRID:CVCL_0152) and BxPC3 (RRID:CVCL_0186) cells were maintained in RPMI 1640 Medium (Corning). L3.6pl (RRID:CVCL_0384) and HPAFII (RRID:CVCL_0313) cells were maintained in phenol red-free minimum essential media (MEM; Thermo Fischer Scientific). Panc1 cells were maintained in Dulbecco’s Modified Eagle Medium (DMEM; Corning). Media were supplemented with 10% FBS (Corning; 20% for Panc1), 1% Penicillin/streptomycin (Thermo Fischer Scientific), and 1% L-Glutamine (Corning, for MEM media). Cells were split upon reaching 70-80% confluence. All treatments were performed in the appropriate media and the list of proteins and inhibitors and concentrations used are provided in Supplementary Table S1. Cells were treated with EGF (10ng/ml; R&D systems; AFL236-200), TNF (10ng/ml; R&D systems 210-TA-100), BI 653048 (300nM; Opnme), and Dexamethasone (30nM; Hozel Diagnostika; HY-14648) Knockdown by siRNA transfections was performed using Lipofectamine RNAiMAX (Thermo Fischer; 56532) following the manufacturer’s recommendations. siGENOME SMARTpool siRNA (Dharmacon) was used for gene depletion. The sequences of the siRNAs used is provided in Supplementary Table S1.

Transfections were carried out using Lipofectamine 3000 (Thermo Fisher; 2429223) according to the manufacturer’s protocol. A total of 2.5 x 10⁵ cells were seeded into each well of six-well plates one day prior to transfection. The cells were then transfected with 2 µg of mutant FOSL1 (S252D_S265D_hFOSL1_3XHA_mCherry_pGenLenti; GenScript), 2 µg of mutant RELA (S536D_hRELA_3Xflag_EGFP_pGenLenti; GenScript), 1 µg of mCherry (pcDNA4.1/mycHis/RfB-mCherry), and 1 µg of EGFP (pRGFP-C1; Takara Bio). After 24 hours of transfection, the cells were passaged for IncuCyte time-lapse imaging and western blot analysis.

**Protein isolation, RNA isolation, quantitative real-time PCR (qPCR), RNA-seq and ChIP-seq library preparation**

Protein isolation and western blots were performed as previously reported [3]. RNA was extracted using QIAzol reagent (Qiagen, Venlo, Netherlands). Antibodies and primers used are in this study are in Tables S2 and S3 respectively. Relative mRNA expression was normalized to vehicle and siNT5 controls. qPCR data are shown as mean ± s.d. from three biological replicates, plotted using grouped or column graphs in GraphPad Prism.

For the RNA-seq library, RNA quality was validated by gel electrophoresis. We used 500ng to make the libraries in triplicates for each condition. Libraries for cells were made using the TruSeq RNA Library Prep Kit V3 (Illumina) according to the manufacturer’s instructions. ChIP sand ChIP-seq were performed as previously described with minor changes [4, 5]. Libraries were prepared using the MicroPlex Library Preparation Kit v2 (Diagenode) according to the manufacturer’s protocol. Details protocol for RNA- and ChIP-seq are provided in Supplementary information. DNA quality of the resulting DNA was measured using the High Sensitivity DNA Kit (Agilent) on the Agilent TapeStation 4150 (RRID:SCR_019393). Antibodies used for ChIP are provided in the ChIP-seq methods section below. Samples were sequenced (paired-end 50 bp) on a HiSeq 4000 (Illumina; RRID:SCR_016386) at the Genome Analysis Core at the Mayo Clinic (RRID:SCR_024632; for 30-minute-treated RNA-seq samples) and on a NextSeq 2000 (P2, Illumina; RRID:SCR_023614) at the Robert Bosch Center for Tumor Diseases (RBCT) (for 48-hour-treated RNA-seq samples).

**scRNAseq data analysis for publicly available datasets**

For the single cell RNAseq analysis, the following publicly available PDAC patient datasets were downloaded from the GEO database (RRID:SCR_005012), GSE154778 [6], GSE111672 [7], PRJCA001063 [8], GSE155698 [1], GSM4293555 from GSE141017 [9], and scRNA-seq data from the study by Chijimatsu et al., 2022 [2]. The processed data for PRJCA001063 [8] was obtained from zenodo [10.5281/zenodo.3969339] (RRID:SCR_004129), which included cell label annotations for 10 cell types after quality control (QC) steps. Other scRNA-seq datasets were downloaded from the NCBI GEO database or as specified in their respective publications. These datasets were combined and prepared for downstream analysis using the bioinformatics methodology described by Chijimatsu et al., 2022 [2].

Seurat objects (RRID:SCR_022555) were created for individual datasets by reading the Cellranger output files into the R environment using the Read10x function and transforming them using the CreateSeuratObject function, as described in the Seurat Guided Clustering Tutorial (<https://satijalab.org/seurat/articles/pbmc3k_tutorial>). The analysis was conducted using R version 4.2.3 and Seurat version 4.3.0.1. Transcript counts, measured as UMIs, were normalized to 10,000 counts per cell and log-transformed, following the methodology described by Chijimatsu et al., 2022 [2]. Cells with a high percentage of mitochondrial genes (>25%) were filtered out during the QC steps. Other QC metrics for individual datasets, such as UMI counts and the number of expressed genes, were also applied as per Chijimatsu et al., 2022 [2].

Datasets were batch-corrected and integrated using the rPCA method as outlined in the Seurat package (<https://satijalab.org/seurat/articles/integration_rpca.html>). Each dataset was scaled, and the FindVariableFeatures function was employed to identify highly variable genes. These genes were utilized for PCA analysis (RRID:SCR_014676). An anchor was created using the FindIntegrationAnchors function with the following parameters: 30 principal components, rPCA, and two reference datasets (PRJCA001063 and GSE155698). Subsequently, six datasets were integrated using the IntegrateData function of the Seurat package. The integrated dataset was scaled, followed by PCA analysis and UMAP (RRID:SCR_018217) visualization. Cell-type annotation was transferred from the reference dataset PRJCA001063 [8].

**Cell migration analysis from incuCyte time-lapse imaging**

A total of 3000 cells were seeded and treated overnight with Nuclight Red dye (1:2000; Sartorius). The cells were then treated with proteins and inhibitors as specified in the cell culture section above. Live cell imaging was performed using the Sartorius IncuCyte (RRID:SCR_023147). Images were captured every 15 minutes for 48 hours and processed using the Basic Analyzer tool (Sartorius).

Preprocessing and image analysis were performed using FIJI (RRID:SCR_002285) [10]. Raw brightfield and fluorescent nuclear tiff images from IncuCyte imaging were compiled using a custom script written in the ImageJ (RRID:SCR_003070) macro language [11]. Imaging drift was corrected in batch using the Correct 3D Drift plugin [12]. Nuclear segmentation and tracking were performed in batches using a custom Python (RRID:SCR_008394) script in ImageJ. Image nuclei were automatically segmented for each imaging frame using the StarDist2D ImageJ plugin with the included “Versatile (fluorescent nuclei)” model [13, 14]. The resulting labeled images were then tracked frame-by-frame in a semi-automated manner using the TrackMate plugin for ImageJ with a Simple Sparse LAP Tracker and a maximum linking distance and gap closing distance of 50 px [15]. The resulting cell trajectories were output to an xml file and further analyzed using custom Python (v3.9) software utilizing the following packages: numpy (RRID:SCR_008633), scipy (RRID:SCR_008058), pandas (RRID:SCR_018214), and matplotlib (RRID:SCR_008624) [16-19]. Tracks containing <3 time points were disregarded. Mean instantaneous speeds, mean squared displacements (MSDs), and directional correlation between trajectories were calculated as previously described [20]. Data from different conditions was compiled, and custom Python scripts were used to perform statistical analysis and visualization.

Box plots were plotted using the 10th to 90th percentile range. The error bars represent the 10th and 90th percentiles of the data, reflecting the spread of the central 80% of values. These are percentile-based and do not correspond to standard deviation (s.d.), standard error of the mean (s.e.m.), or confidence intervals (c.i.).

**Cellular transwell assay to test cell migration**

Approximately 50,000 AsPC-1 and L3.6pl cells were uniformly seeded onto the upper layer of the transwell membrane with an 8-μm pore size (Corning). The cells were treated with the appropriate medium containing the necessary treatments. After incubation at 37°C for 96 hours, the cells that migrated through the membrane were collected for RNA isolation and assessed using crystal violet staining.

For the co-culture assays, between 300,000 and 500,000 THP-1 cells were plated at the bottom of a 24-well insert plate (Corning) in serum-free media and stimulated with 0.1 nM phorbol 12-myristate-13-acetate (PMA) to induce differentiation into macrophage-like cells over 48 hours. The conditioned media was then collected, centrifuged, and mixed with normal media in a 3:1 ratio, and this mixture was used, along with the treatments, for the migration assay as described above.

**mRNA-seq**

The integrity of RNA was validated by gel electrophoresis and 500ng was used to make the libraries in triplicates for each condition. Libraries for cells were made using the TruSeq RNA Library Prep Kit V3 (Illumina) according to the manufacturer’s instructions. Oligo-dT beads were used to capture poly-A tailed-mRNA followed by first-strand cDNA synthesis by Superscript II reverse transcriptase (Thermo Fischer). Second-strand synthesis was followed by end repair, 3’ adenylation, adaptor ligation, and library amplification. Agencourt AMPure XP (Beckman Coulter) was used for size selection during the library synthesis. The quality of the resulting DNA was measured with high sensitivity DNA kit (Agilent) on the Agilent TapeStation 4150. Samples were sequenced (paired-end 50bp) on a HiSeq4000 (Illumina) at the Genome Analysis Core at the Mayo Clinic (30-minute-treated RNA-seq samples) and on a NextSeq 2000 (P2, Illumina) at the Robert Bosch Center for Tumor diseases (RBCT).

**RNA-seq bioinformatic analysis**

Bam files were generated using STAR version 2.7.3a (RRID:SCR_004463) [21]. Features were counted using htseq version 0.9.1 (RRID:SCR_005514) [22]. Differential gene expression analysis was performed by DESeq2 (RRID:SCR_015687) [23]. Gene set enrichment analysis (GSEA; RRID:SCR_003199) [24] was performed with default setting using normalized counts from DESeq2 for expressed genes. Upregulated genes were identified as ≥1 log2 Fold Change, FDR≤0.05, and BaseMean≥10 (48-hour RNAseq) and ≥0.5 log2 Fold Change, FDR≤0.05, and BaseMean≥10 (30-minute RNAseq). Pathway analyses were performed using ShinyGO version 0.80 (RRID:SCR_019213) [25].

**RNA-seq Quality Control and Sample Exclusion**

For both AsPC-1 (Fig. 2b) and L3.6pl (Fig. S2b) cell lines, one RNA-seq sample from the TNFα-treated group (replicate 1) was excluded from downstream analysis. This decision was based on pre-analysis quality control, which included evaluation of read counts, principal component analysis (PCA), and z-score heatmaps. In both cases, the excluded sample exhibited markedly low total read counts and clustered as an outlier in PCA plots. Further investigation revealed the issue was due to sequencing error. Although the exclusion criteria were not pre-established, they were applied consistently based on these objective quality metrics.

**Chromatin immunoprecipitation sequencing (ChIP-seq)**

Chromatin immunoprecipitation was performed as previously described [5, 26]. Antibodies included H3K27ac (1μg; C15410196, Diagenode; RRID:AB_2637079), FOSL1 (D80B4) (FRA-1) (5 μl; 5281; Cell Signaling; RRID:AB_10557418), RELA (NFκB p65 L8F6) (5 μl; 6956; Cell Signaling; RRID:AB_10828935), RNAPII (Rpbl NTD D8L4Y) (5 μl; 14958; Cell Signaling; RRID:AB_2687876), NR3C1 (7.5 μg; C15200010, Diagenode; RRID:AB_2801409), and control Rabbit IgG (1 μg; C15410206, Diagenode; RRID:AB_2722554). Protein A-sepharose and protein G-Sepharose (for RELA) beads were added to the samples and incubated for 2 hours, washed, de-crosslinked, and DNA was extracted. Samples were performed in triplicates for each. Condition. Libraries were prepared using the MicroPlex Library Preparation Kit v2 (Diagenode) according to the manufacturer’s protocol. DNA integrity was measured with a high-sensitivity DNA kit (Agilent) on the Agilent TapeStation 4150. Samples were sequenced (paired-end 50bp) on a HiSeq4000 (Illumina) at the Genome Analysis Core at the Mayo Clinic (30-minute-treated RNA-seq samples) and on a NextSeq 2000 (P3, Illumina) at the Robert Bosch Center for Tumor diseases (RBCT) (48-hour-treated RNA-seq samples).

**ChIP-seq bioinformatic analysis**

Reads were mapped to the reference genome assembly (hg38) by BOWTIE2/2.5.0 (RRID:SCR_016368) [27]. Bigwig files were generated from merged bam files using bamCoverage (RRID:SCR_016366). Localization profiles were viewed using Integrative Genomics Viewer (IGV 2.16.0) (RRID:SCR_011793) [28]. MACS2 (RRID:SCR_013291) [29] was used to call the significant peaks without building the shifting model with broad peaks (broad-cutoff 0.05) called for H3K27ac, narrow peaks (broad-cutoff 0.05), and input files from respective cells as background. The bigwigs for L3.6pl H3K27ac ChIP-seq were normalized using the Spike-In Free ChIP-seq normalization approach [30]. The Bioconductor (RRID:SCR_006442) R package Diffbind 3.10.1 (RRID:SCR_012918) [31] was run on R version 4.3.1 according to the instruction manual to define regions that are differentially enriched by H3K27ac. ChIP occupancies were evaluated by the computeMatrix tool (RRID:SCR_016366). ChIP-seq profiles and heatmaps were generated from computeMatrix values using the PlotProfiles and PlotHeatmap tools respectively on the Galaxy platform (RRID:SCR_006281) [32]. Nearby genes on genomic regions were annotated using the Genomic Regions Enrichment of Annotations (GREAT) tool (version 4.0.4) (RRID:SCR_005807) [33, 34]. Transcription factor enrichment analyses were performed using ChIP-Atlas (RRID:SCR_015511) [35].

For NR3C1 data in Supplemental Figure 5b, a DiffBind analysis of H3K27ac regions was performed after combined treatment versus vehicle. Regions showing a log fold >/= 1 in the combined treatment were selected and intersected (using bedtools intersect) with NR3C1 regions from the ChIP-Atlas database. A ChIP-Atlas analysis was then conducted to identify enriched transcription factors within those regions.

**Immunofluorescence**

Cells were fixed on glass slides with 4% paraformaldehyde (ROTI Histofix) in PBS for 10 minutes, washed twice with 0.1% Triton X-100 in PBS with a 20-minute incubation, and washed with PBS. The cells were then blocked with 1% BSA in PBS for 30 minutes and incubated with primary antibody overnight in the dark at 4^o^C 1:500. Primary antibodies applied were mouse anti-FRA-1 C-12 (FOSL1; Santa Cruz; sc-28310; RRID:AB_627632) and rabbit anti-NFκB p65 (L8F6) (RELA; Cell Signaling; 6956; RRID:AB_10828935). Following incubation, cells were rinsed with PBS and treated with secondary antibodies in blocking solution for 1 hour at room temperature. The secondary antibodies used are Alexa Fluor 596 anti-mouse IgG (Invitrogen; A21201; RRID:AB_2535787) and Alexa Fluor 488 anti-rabbit IgG (Invitrogen; A21441; RRID:AB_2535859). Cells were rinsed again and stained with the Duolink *In Situ* mounting medium containing DAPI (Sigma Aldrich; SLCR3027). Cells were imaged under a fluorescence microscope (Leica Microsystems TCS SP8; RRID:SCR_024563) equipped with filters for DAPI (377nm), FITC (485nm), and Cy3 (560nm).

**Proximity ligation assay (PLA)**

PLA assay was performed according to the manufacturer’s protocol (link; chrome-extension://efaidnbmnnnibpcajpcglclefindmkaj/https://www.sigmaaldrich.com/deepweb/assets/sigmaaldrich/marketing/global/documents/133/014/duolink-pla-fluorescence-protocol-5325-mk.pdf). Primary antibodies applied were mouse anti-FRA-1 C-12 (FOSL1; Santa Cruz; sc-28310; RRID:AB_627632) and rabbit anti-NFκB p65 (L8F6) (RELA; Cell Signaling; 6956; RRID:AB_10828935). Cells stained with the Duolink In Situ mounting medium containing DAPI (Sigma Aldrich; SLCR3027). Cells were imaged under a fluorescence microscope (Leica Microsystems TCS SP8; RRID:SCR_024563) equipped with filters for DAPI (377nm), FITC (485nm), and Cy3 (560nm).

Box plots show the distribution of PLA signal per nucleus. The box spans the interquartile range with the median indicated, and whiskers represent the 10th and 90th percentiles (or as defined by Tukey's method). Data do not represent mean ± s.d./s.e.m.

**Multiplex immunofluorescence staining**

A six-color multiplex immunofluorescence staining was performed using OPAL^TM^ multiplexing method. The staining protocol for FFPE tissue sections was optimized for the simultaneous detection of 6 antibodies and DAPI for cell nuclear stain. The sections were deparaffinized, rehydrated, subjected to heat-induced epitope retrieval, and incubated with primary and secondary antibodies. The antibodies were visualized using a fluorescent tyramide with Opal 6-Plex Manual Detection Kit (Akoya Biosciences; NEL861001KT). The epitope retrieval and staining process was repeated sequentially for different primary antibodies and fluorescent tyramide combinations. The following primary antibodies with different dilutions were used: CD68 (Cell Marque; 168M-94; RRID:AB_1158188) with 1:500 dilution, IL-1β (3A6) (Cell Signaling; 12242; RRID:AB_2715503) with 1:10 dilution, Phospho-NF-kB p65 (Ser536) (Cell Signaling; 3033; RRID:AB_331284) with 1:25 dilution, αSMA (Abcam; ab5694; RRID:AB_2223021) with 1:100 dilution, and Pan-Keratin (AE1/AE3) (Cell Signaling; 67306) with 1:50 dilution. Antibodies were visualized with the following tyramide dyes used from the Opal Detection kit (Akoya Biosciences; NEL861001KT): Opal 520, Opal 570, Opal 620, Opal 690, and DIG-Opal 780. Sections were mounted with ProLong^®^ Diamond Antifade Mountant (Thermo Fischer Scientific; P36961). Multiplex-stained slides were imaged using a PhenoImager Fusion system (Akoya Biosciences).

**Cell-derived orthotopic xenograft establishment and treatment**

All planned experiments adhered to the National Institutes of Health Guide for the Care and Use of Laboratory Animals. The Institutional Animal Care and Use Committee (IACUC) unequivocally approved the animal protocol at the Mayo Clinic (#A00003954-18-R21). The animal care facilities and use program consistently comply with all federal regulations and guidelines. Mayo Foundation is proudly registered with the USDA (41-R-006) as an animal research facility and maintains an NIH animal assurance statement (A3291-01) with the Office of Laboratory Animal Welfare.

The corresponding authors, who conceptualized and designed the study, were intentionally kept unaware of the group allocation at different stages of the study. The studies were conducted explicitly in an independent PDX laboratory, where the laboratory scientists and personnel were not blinded to the conduct of the experiments, assessment, or data analyses.

The research in this manuscript involved a hairless (albino) Athymic Nude mouse (Crl:NU(NCr)-*Foxn1^nu^* Immunodeficient Outbred; strain code number: 490) female mouse obtained from Charles River Laboratory (Charles River, USA). The mouse was fed LabDiet PicoLab Rodent Diet 20 (Lab Supply, Fort Worth, Texas) and housed in an Innovative Disposable caging system called Innocage® Mouse Pre-Bedded Corn Cob, in the validated Innorack® IVC Mouse 3.5 (InnoVive, San Diego, California). Five mice were housed per cage in a 12-hour light/dark cycle with continuous access to food and water and no fasting. The number of mice per group was minimized to 5 mice per group to reduce the use of laboratory mice, considering these as early feasibility/pilot in vivo studies (as per ARRIVE Guidelines).

The luciferase-tagged cultured cells underwent trypsinization and neutralization, after which the cell suspension was carefully transferred to a separate 50 ml falcon tube. Following a precise cell count, we meticulously prepared a sufficient number of cells (1x10^6^ cells in 1.5 mL of suspension media and Matrigel in a 1:1 ratio) for orthotopic injection, ensuring 15 mice per cell type could be accommodated. Throughout this process, the cell suspension was kept on ice to prevent Matrigel solidification.

Prior to the procedure, the surgical site was shaved, and the mice were thoroughly anesthetized and provided with appropriate analgesia. Furthermore, the surgical site was meticulously cleaned with a sterile 3 ml ChloraPrep^TM^ Hi-Lite Orange^TM^ applicator. Subsequently, minor 1 cm oblique surgical incisions were carefully made on each mouse's left subcostal area to enable access.

The pancreas and spleen were identified using moistened cotton swabs, ensuring aseptic conditions were maintained. Lift and separation of the spleen and pancreas from the abdominal cavity were then achieved using the cotton swab. Notably, great care was taken to prevent cell lysis during the subsequent steps. A 25G needle was skillfully inserted into the tail of the pancreas and passed into the pancreatic head region. With precision, 30-40 uL of the solution was slowly injected using a 25G needle while simultaneously withdrawing the needle to the mid-body, generating a distinct fluid-filled region within the parenchyma. After the injection, we meticulously checked for bleeding and leaks. Following this, the pancreas was left externalized and untouched for 2 minutes to allow the Matrigel to solidify. The subsequent gentle internalization of the abdominal contents was executed with the utmost care and precision.

The muscle and skin layers were closed separately, utilizing running 3-0 Chromic sutures. Post-procedure, the mice were diligently recovered under a heat lamp until fully awake. Notably, a cell line type was implanted into fifteen mice, accounting for any additional mice that might be required for the experiment.

Luciferase-tagged cell lines have been observed to exhibit rapid growth, prompting their treatment one week after implantation into the pancreas of nude mice. Following implantation, the mice were allocated into two groups (5 mice/group) based on tumor volume: 1) Vehicle; 2) BI653048 (30 mg/kg) oral gavage (OG) daily. Their treatment period was approximately four weeks, during which IVIS imaging was conducted biweekly. On the 28th day of treatment or upon the natural death of the mice, euthanasia was performed using carbon dioxide administered by IACUC-trained personnel, followed by cervical dislocation.

**Human subjects’ compliance statement:**

All human tumor samples used for multiplex immunofluorescence were obtained through the Mayo Clinic Institutional Patient-Derived Xenograft (PDX) program for hepatobiliary, pancreatic tumors, and aggressive abdominal malignancies. Collection of these samples was conducted under approved Mayo Clinic Institutional Review Board (IRB) protocols (66-06, 354-06, 19-012104). All procedures involving human specimens complied with institutional guidelines and the Declaration of Helsinki.

**Immunohistochemical staining (IHC)**

Paraffin-embedded tissue sections were deparaffinized in 100% Neoclear, rehydrated through a series of graded ethanol solutions (100%-70%), and rinsed in dH_2_O. All buffers used were from the Opal 6-plex Manual Detection kit (Akoya Biosciences; NEL861001KT). Antigen retrieval was conducted by incubating the slides in AR6 buffer and heating them for 30 minutes in a steamer. After allowing the slides to cool for 20 minutes, the sections were washed in 1X TBST. The sections were then incubated with 1X antibody diluent/block buffer for 30 minutes, washed, and incubated with pan cytokeratin antibody (1:300, Abcam; ab217916). After washes with TBST, sections were incubated with Anti-rabbit IgG, HRP-linked (Cell Signaling; 7074S; RRID:AB_2099233) for 1 hour. Following a final wash in 1X TBST, the sections were developed using the DAB kit (1:50, Agilent Technologies; K500711-2). The sections were stained with hematoxylin (Sigma Aldrich; HX43732253), dehydrated with graded ethanol and Neoclear, and finally mounted using Neo-Mount (Sigma Aldrich; HX29658016). Scans were captured using the Olympus Upright microscope (BX61VS).

**Tables**

Table S1: Sequences of siRNAs used in this study

| Non-targeting #5 (NT5) | UGGUUUACAUGUCGACUAA |
| --- | --- |
| FOS | Dharmacon; MQ-004341-04-0010 |
| FOS, FOSL1, JUND | Dharmacon; G-CUSTOM-732397 (AP1 factors); RNAi cherry pick library |
| NFκB1, NFκB2, RELA, and RELB | Dharmacon; G-CUSTOM-732397 (NF-κB transcription factors); RNAi cherry pick library |
| ATF3 | Dharmacon; G-CUSTOM-732397 (MAFF transcription factors); RNAi cherry pick library |

Table S2: Antibodies used in this study.

| **Antibodies** | **Company; Catalogue number** |
| --- | --- |
| P-p44/42 MAPK (T202/Y204) | Cell Signaling; 4370; RRID:AB_2315112 |
| p44/42 MAPK (ERK1/2) (3A7) | Cell Signaling; 9107; RRID:AB_10695739 |
| P-MEK1/2 (S217/221) (41G9) | Cell Signaling; 9154; RRID:AB_2138017 |
| MEK1/2 (L38C12) | Cell Signaling; 4694; RRID:AB_10695868 |
| P-NFκB p65 (S536) (93H1): phospho-RELA | Cell Signaling; 3033; RRID:AB_331284 |
| NFκB p65 (L8F6) | Cell Signaling; 6956; RRID:AB_10828935 |
| FRA-1 C-12 (FOSL1) | Santa Cruz; sc-28310; RRID:AB_627632 |
| P-IKKα/β (S176/180) (16A6) | Cell Signaling; 2697; RRID:AB_2079382 |
| IKKα (3G12) | Cell Signaling; 11930; RRID:AB_2687618 |
| β-Actin (D6A8) | Cell Signaling; 8457; RRID:AB_10950489 |
| HSP70 | Cell Signaling; 4872; RRID:AB_2279841 |
| **Secondary Antibodies** |  |
| Goat anti-rabbit IgG Starbright Blue 700 | BioRad; 1200416; RRID:AB_2721073 |
| Goat anti-mouse IgG Starbright Blue 520 | BioRad; 12005866; RRID:AB_2934034 |

Table S3: Primers in this study.

| **Gene** | **Forward sequence (5’-3’)** | **Reverse sequence (5’-3’)** |
| --- | --- | --- |
| *CXCL8* | CGGAAGGAACCATCTCACTGTG | AGAAATCAGGAAGGCTGCCAAG |
| *IL1β* | CCACAGACCTTCCAGGAGAATG | GTGCAGTTCAGTGATCGTACAGG |
| *IL1α* | TGTATGTGACTGCCCAAGATGAAG | AGAGGAGGTTGGTCTCACTACC |
| *IGFBP6* | GACTGAGGTCTACCGAGGGG | AGGGCATTCCTTAAGTGGGTT |
| *MMP10* | AATAGCCAGTCCATGGAGCAA | TAAGGGTCTTCTTACCAAATGCCT |
| *MMP1* | GGCCACAAAGTTGATGCAGT | TTGCTACGGCAATGAAATGGAG |
| *LAMC2* | CCACGTTGAGTCAGCCAGTA | GGAGGACCCGTAGGAACGTA |
| *TNFAIP3* | AGGACAGAAGAGCAACTGGTG | AACGCTCCAGCAAAAAGCATC |
| *TNF* | CTCTTCTGCCTGCTGCACTTTG | ATGGGCTACAGGCTTGTCACTC |
| **ChIP-qPCR Primers** |  |  |
| FOSL1_RELA_co-bound at *IL1B* | GTATCAAGCAATTGCAAACTTCCTG | ATTCACTTTGTCTCCACCAGAACTC |

Table S4: Metastasis-related genes identified in Figure 1i and S1c and f.

| Genes | References |
| --- | --- |
| *LCN2* | Gomez-Chou, S.B., et al., *Lipocalin-2 Promotes Pancreatic Ductal Adenocarcinoma by Regulating Inflammation in the Tumor Microenvironment.* Cancer Res, 2017. **77**(10): p. 2647-2660. |
| *ERO1L* | Yang, J., et al. (2021). "ERO1L Promotes Hepatic Metastasis through Activating Epithelial-Mesenchymal Transition (EMT) in Pancreatic Cancer." J Immunol Res **2021**: 5553425. |
| *SEP15* | Bang, J., et al. (2015). "Cell Proliferation and Motility Are Inhibited by G1 Phase Arrest in 15-kDa Selenoprotein-Deficient Chang Liver Cells." Mol Cells **38**(5): 457-465. |
| *PSCA* | Li, X. P., et al. (2015). "Expression of CD44 in pancreatic cancer and its significance." Int J Clin Exp Pathol **8**(6): 6724-6731. |
|  | Nayerpour Dizaj, T., et al. (2024). "Significance of PSCA as a novel prognostic marker and therapeutic target for cancer." Cancer Cell Int **24**(1): 135. |
| *IL8* | Fang, Y., et al. (2016). "IL-8-Positive Tumor-Infiltrating Inflammatory Cells Are a Novel Prognostic Marker in Pancreatic Ductal Adenocarcinoma Patients." Pancreas **45**(5): 671-678. |
|  | Cheng, Y., et al. (2019). "Potential roles and targeted therapy of the CXCLs/CXCR2 axis in cancer and inflammatory diseases." Biochim Biophys Acta Rev Cancer **1871**(2): 289-312. |
| *DKK1* | Liu, D. J., et al. (2017). "The role of Dickkopf-1 as a potential prognostic marker in pancreatic ductal adenocarcinoma." Cell Cycle **16**(17): 1622-1629. |
|  | Baarsma, H. A., et al. (2013). "The WNT signaling pathway from ligand secretion to gene transcription: molecular mechanisms and pharmacological targets." Pharmacol Ther **138**(1): 66-83. |
|  | Jiang, H., et al. (2014). "Activation of the Wnt pathway through Wnt2 promotes metastasis in pancreatic cancer." Am J Cancer Res **4**(5): 537-544. |
| *RARRES3* | Wu M, Li X, Liu R, Yuan H, Liu W, Liu Z. Development and validation of a metastasis-related Gene Signature for predicting the Overall Survival in patients with Pancreatic Ductal Adenocarcinoma. *J Cancer* 2020; 11(21):6299-6318. |
| *HN1* | Zhang, C., et al. (2017). "HN1 contributes to migration, invasion, and tumorigenesis of breast cancer by enhancing MYC activity." Mol Cancer **16**(1): 90. |
|  | Niu, Z., et al. (2015). "Knockdown of c-Myc inhibits cell proliferation by negatively regulating the Cdk/Rb/E2F pathway in nasopharyngeal carcinoma cells." Acta Biochim Biophys Sin (Shanghai) **47**(3): 183-191. |
| *NEAT1* | Cao, J., et al. (2016). "NEAT1 regulates pancreatic cancer cell growth, invasion and migration though mircroRNA-335-5p/c-met axis." Am J Cancer Res **6**(10): 2361-2374. |
|  | Feng, Y., et al. (2020). "LncRNA NEAT1 facilitates pancreatic cancer growth and metastasis through stabilizing ELF3 mRNA." Am J Cancer Res **10**(1): 237-248. |
|  | Huang, B., et al. (2017). "Long non-coding RNA NEAT1 facilitates pancreatic cancer progression through negative modulation of miR-506-3p." Biochem Biophys Res Commun **482**(4): 828-834. |
| *PLCG2* | Li, Z., et al. (2021). "PLCG2 as a potential indicator of tumor microenvironment remodeling in soft tissue sarcoma." Medicine (Baltimore) **100**(11): e25008. |
| *SNCG* | Bruening, W., et al. (2000). "Synucleins are expressed in the majority of breast and ovarian carcinomas and in preneoplastic lesions of the ovary." Cancer **88**(9): 2154-2163.  Liu, H., et al. (2005). "Loss of epigenetic control of synuclein-gamma gene as a molecular indicator of metastasis in a wide range of human cancers." Cancer Res **65**(17): 7635-7643. |
|  | Liu, J., et al. (2022). "Gamma synuclein promotes cancer metastasis through the MKK3/6-p38MAPK cascade." Int J Biol Sci **18**(8): 3167-3177. |
| *SEPP1* | Marciel, M. P. and P. R. Hoffmann (2017). "Selenoproteins and Metastasis." Adv Cancer Res **136**: 85-108. |

**References**

1. Steele, N.G., et al., *Multimodal Mapping of the Tumor and Peripheral Blood Immune Landscape in Human Pancreatic Cancer.* Nat Cancer, 2020. **1**(11): p. 1097-1112.

2. Chijimatsu, R., et al., *Establishment of a reference single-cell RNA sequencing dataset for human pancreatic adenocarcinoma.* iScience, 2022. **25**(8): p. 104659.

3. Kutschat, A.P., et al., *STIM1 Mediates Calcium-Dependent Epigenetic Reprogramming in Pancreatic Cancer.* Cancer Res, 2021. **81**(11): p. 2943-2955.

4. Najafova, Z., et al., *BRD4 localization to lineage-specific enhancers is associated with a distinct transcription factor repertoire.* Nucleic Acids Res, 2017. **45**(1): p. 127-141.

5. Hamdan, F.H. and S.A. Johnsen, *DeltaNp63-dependent super enhancers define molecular identity in pancreatic cancer by an interconnected transcription factor network.* Proc Natl Acad Sci U S A, 2018. **115**(52): p. E12343-E12352.

6. Lin, W., et al., *Single-cell transcriptome analysis of tumor and stromal compartments of pancreatic ductal adenocarcinoma primary tumors and metastatic lesions.* Genome Med, 2020. **12**(1): p. 80.

7. Moncada, R., et al., *Integrating microarray-based spatial transcriptomics and single-cell RNA-seq reveals tissue architecture in pancreatic ductal adenocarcinomas.* Nat Biotechnol, 2020. **38**(3): p. 333-342.

8. Peng, J., et al., *Single-cell RNA-seq highlights intra-tumoral heterogeneity and malignant progression in pancreatic ductal adenocarcinoma.* Cell Res, 2019. **29**(9): p. 725-738.

9. Schlesinger, Y., et al., *Single-cell transcriptomes of pancreatic preinvasive lesions and cancer reveal acinar metaplastic cells' heterogeneity.* Nat Commun, 2020. **11**(1): p. 4516.

10. Schindelin, J., et al., *Fiji: an open-source platform for biological-image analysis.* Nat Methods, 2012. **9**(7): p. 676-82.

11. Rasband, W.S., *ImageJ [Online].* <https://imagej.net/>, 1997.

12. Parslow, A., A. Cardona, and R.J. Bryson-Richardson, *Sample drift correction following 4D confocal time-lapse imaging.* J Vis Exp, 2014(86).

13. Uwe Schmidt, M.W., Coleman Broaddus & Gene Myers *Cell Detection with Star-Convex Polygons BT Medical Image Computing and Computer Assisted Intervention – MICCAI* 2018: p. 265–273.

14. Weigert, M.S., U.;Haase, R.; Sugawara K.; Myers, G., *Star-convex Polyhedra for 3D Object Detection and Segmentation in Microscopy.* IEEE Winter Conference on Applications of Computer Vision (WACV), 2020: p. 3655–3662.

15. Tinevez, J.Y., et al., *TrackMate: An open and extensible platform for single-particle tracking.* Methods, 2017. **115**: p. 80-90.

16. Hunter, J.D., *Matplotlib: A 2D Graphics Environment.* Comput Sci Eng 2007. **9**: p. 90-95.

17. McKinney, W., *Data Structures for Statistical Computing in Python. .* 2010: p. 56–61.

18. Virtanen, P., et al., *SciPy 1.0: fundamental algorithms for scientific computing in Python.* Nat Methods, 2020. **17**(3): p. 261-272.

19. Harris, C.R., et al., *Array programming with NumPy.* Nature, 2020. **585**(7825): p. 357-362.

20. Clark, A.G., et al., *Self-generated gradients steer collective migration on viscoelastic collagen networks.* Nat Mater, 2022. **21**(10): p. 1200-1210.

21. Dobin, A., et al., *STAR: ultrafast universal RNA-seq aligner.* Bioinformatics, 2013. **29**(1): p. 15-21.

22. Anders, S., P.T. Pyl, and W. Huber, *HTSeq--a Python framework to work with high-throughput sequencing data.* Bioinformatics, 2015. **31**(2): p. 166-9.

23. Love, M.I., W. Huber, and S. Anders, *Moderated estimation of fold change and dispersion for RNA-seq data with DESeq2.* Genome Biol, 2014. **15**(12): p. 550.

24. Subramanian, A., et al., *Gene set enrichment analysis: a knowledge-based approach for interpreting genome-wide expression profiles.* Proc Natl Acad Sci U S A, 2005. **102**(43): p. 15545-50.

25. Ge, S.X., D. Jung, and R. Yao, *ShinyGO: a graphical gene-set enrichment tool for animals and plants.* Bioinformatics, 2020. **36**(8): p. 2628-2629.

26. Hamdan, F.H., et al., *Interactive enhancer hubs (iHUBs) mediate transcriptional reprogramming and adaptive resistance in pancreatic cancer.* Gut, 2023. **72**(6): p. 1174-1185.

27. Langmead, B. and S.L. Salzberg, *Fast gapped-read alignment with Bowtie 2.* Nat Methods, 2012. **9**(4): p. 357-9.

28. Robinson, J.T., et al., *Integrative genomics viewer.* Nat Biotechnol, 2011. **29**(1): p. 24-6.

29. Feng, J., et al., *Identifying ChIP-seq enrichment using MACS.* Nat Protoc, 2012. **7**(9): p. 1728-40.

30. Jin, H., et al., *ChIPseqSpikeInFree: a ChIP-seq normalization approach to reveal global changes in histone modifications without spike-in.* Bioinformatics, 2020. **36**(4): p. 1270-1272.

31. Ross-Innes, C.S., et al., *Differential oestrogen receptor binding is associated with clinical outcome in breast cancer.* Nature, 2012. **481**(7381): p. 389-93.

32. Afgan, E., et al., *The Galaxy platform for accessible, reproducible and collaborative biomedical analyses: 2018 update.* Nucleic Acids Res, 2018. **46**(W1): p. W537-W544.

33. McLean, C.Y., et al., *GREAT improves functional interpretation of cis-regulatory regions.* Nat Biotechnol, 2010. **28**(5): p. 495-501.

34. Tanigawa, Y., E.S. Dyer, and G. Bejerano, *Which TF is functionally important in your open chromatin data?* PLoS Comput Biol, 2022. **18**(8): p. e1010378.

35. Oki, S.O., T. *ChIP-Atlas.* 2015; Available from: <https://chip-atlas.org>.
