# Supplementary material for: Therapeutic targeting of FOSL1 and RELA-dependent transcriptional mechanisms to suppress pancreatic cancer metastasis
Source: Cell Death Dis. 2025 Jul 9;16(1):504. doi: 10.1038/s41419-025-07810-x (PMC12241458; doi:10.1038/s41419-025-07810-x)
Supplement: Supplementary file 2 — Supplementary Figure S1 [file 41419_2025_7810_MOESM2_ESM.pdf]

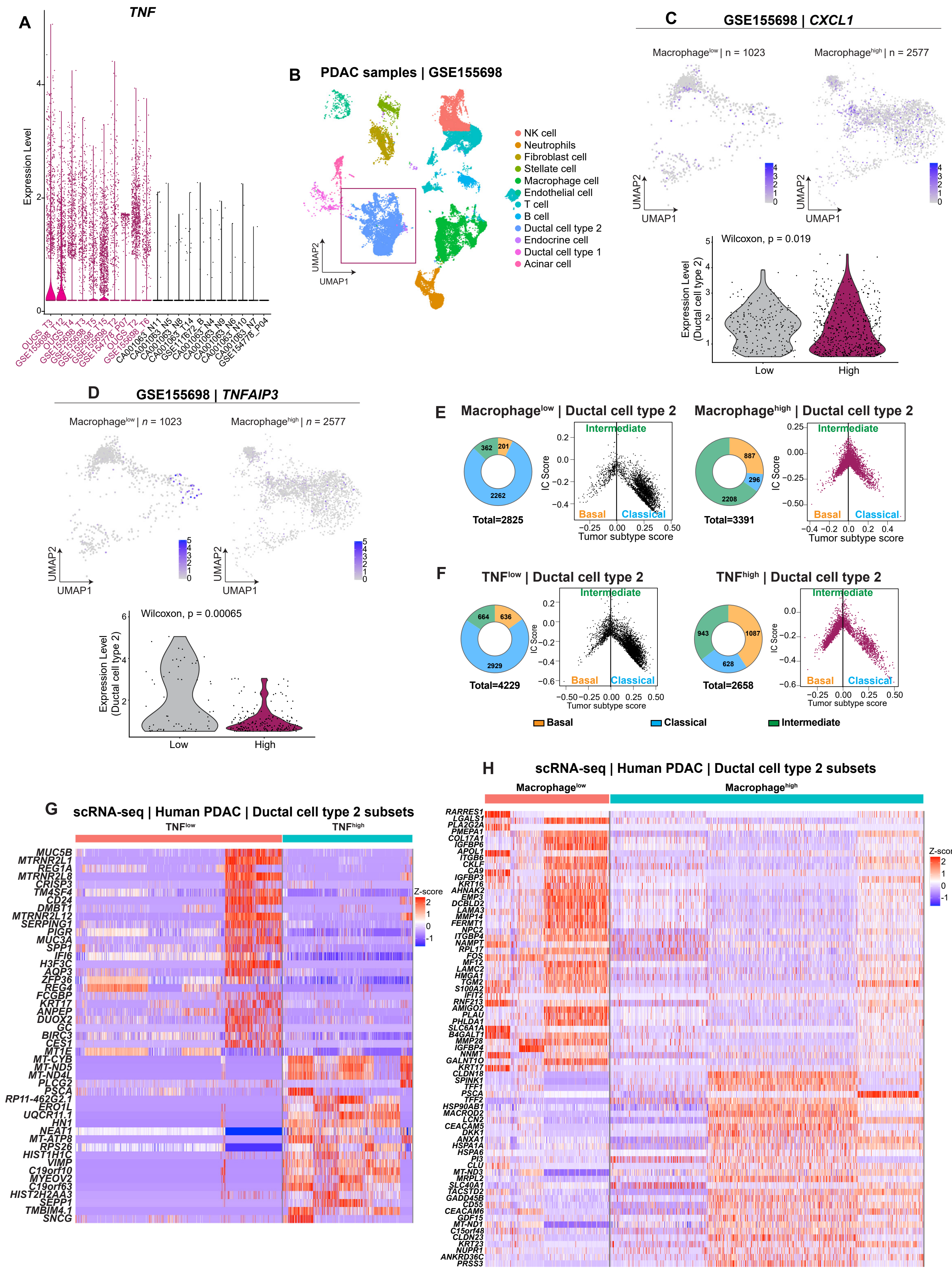
A
TNF
Expression Level
B
PDAC samples | GSE155698
NK cell
Neutrophils
Fibroblast cell
Stellate cell
Macrophage cell
Endothelial cell
T cell
B cell
Ductal cell type 2
Endocrine cell
Ductal cell type 1
Acinar cell
UMAP1
UMAP2
C
GSE155698 | CXCL1
Macrophage^low | n = 1023
Macrophage^high | n = 2577
Wilcoxon, p = 0.019
Expression Level (Ductal cell type 2)
Low
High
D
GSE155698 | TNFAIP3
Macrophage^low | n = 1023
Macrophage^high | n = 2577
Wilcoxon, p = 0.00065
Expression Level (Ductal cell type 2)
Low
High
E
Macrophage^low | Ductal cell type 2
Macrophage^high | Ductal cell type 2
Total=2825
Total=3391
Intermediate
Basal
Classical
IC Score
Tumor subtype score
F
TNF^low | Ductal cell type 2
TNF^high | Ductal cell type 2
Total=4229
Total=2658
Intermediate
Basal
Classical
IC Score
Tumor subtype score
Basal
Classical
Intermediate
G
scRNA-seq | Human PDAC | Ductal cell type 2 subsets
TNF^low
TNF^high
Z-score
MUC5B
MTRNR2L1
REG1A
MTRNR2L8
CRISP3
TM4SF4
CD24
DMBT1
MTRNR2L12
SERPING1
PIGR
MUC3A
SPP1
IFI6
H3F3C
AQP3
ZFP36
REG4
FCGBP
KRT17
ANPEP
DUOX2
GC
BIRC3
CES1
MT1E
MT-CYB
MT-ND5
MT-ND4L
PLCG2
PSCA
RP11-462G2.1
ERO1L
UQCR11.1
HN1
NEAT1
MT-ATP8
RPS26
HIST1H1C
VIMP
C19orf10
MYEOV2
C19orf63
HIST2H2AA3
SEPP1
TMBIM4.1
SNCG
H
scRNA-seq | Human PDAC | Ductal cell type 2 subsets
Macrophage^low
Macrophage^high
Z-score
RARRES1
LGALS1
PLA2G2A
PMEPA1
COL17A1
IGFBP6
APOL1
ITGB6
CKLF
CA9
IGFBP3
KRT16
AHNAK2
EMP3
DCBLD2
LAMA3
MMP14
FERMT1
NPC2
ITGBP4
NAMPT
RPL17
FOS
MF12
LAMC2
HMGA1
TGM2
S100A2
IFIT2
RNF213
AMIGO2
PLAU
PHLDA1
SLC6A14
B4GALT1
MMP28
IGFBP4
NNMT
GALNT10
KRT17
CLDN18
SPINK1
TFF1
PSCA
TFF2
HSP90AB1
MACROD2
LCN2
CEACAM5
DKK1
ANXA1
HSPA1A
HSPA6
PI3
CLU
MT-ND3
MRPL2
SLC40A1
TACSTD2
GADD45B
CD55
CEACAM6
GDF15
MT-ND1
C15orf48
CLDN23
KRT23
NUPR1
ANKRD36C
PRSS3
