# Supplementary figures and images for: Therapeutic targeting of FOSL1 and RELA-dependent transcriptional mechanisms to suppress pancreatic cancer metastasis
Source: Cell Death Dis. 2025 Jul 9;16(1):504. doi: 10.1038/s41419-025-07810-x (PMC12241458; doi:10.1038/s41419-025-07810-x)

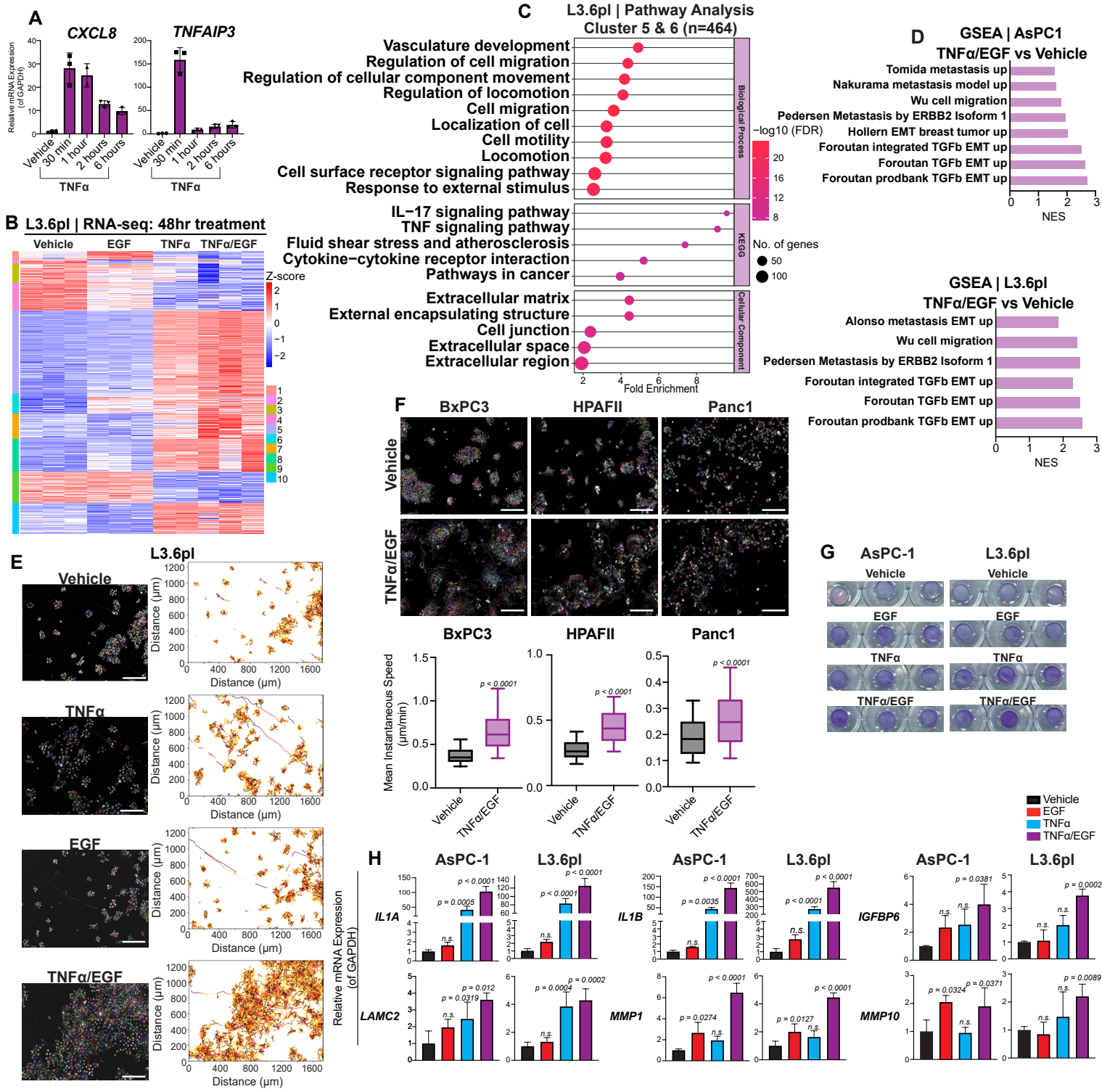

Supplement: Supplementary file 3 — Supplementary Figure S2 [file 41419_2025_7810_MOESM3_ESM.pdf]

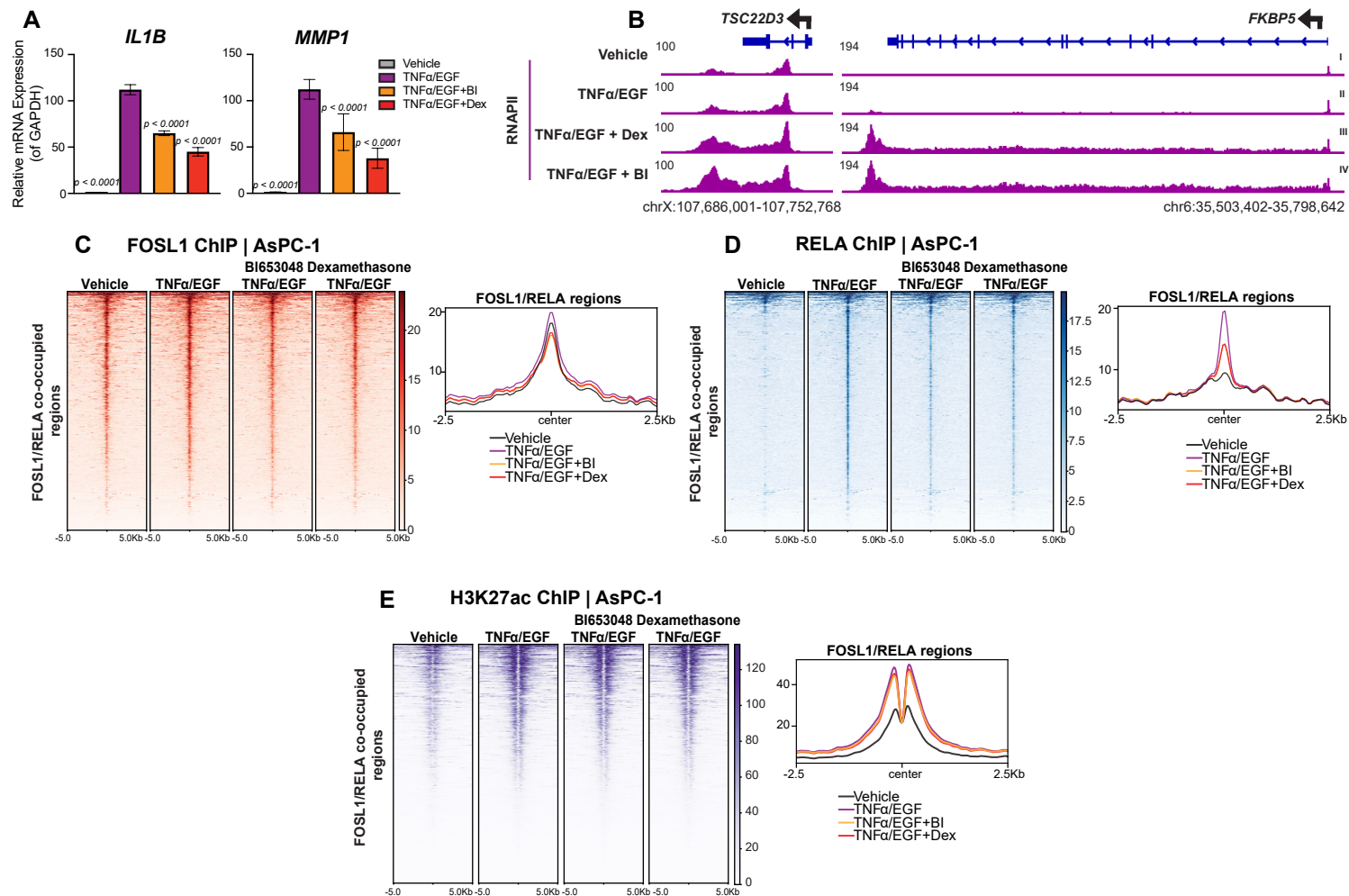

Supplement: Supplementary file 6 — Supplementary Figure S5 [file 41419_2025_7810_MOESM6_ESM.pdf]

Supplementary material 2: western blots used in this study

L3.6pl | Figure 2b

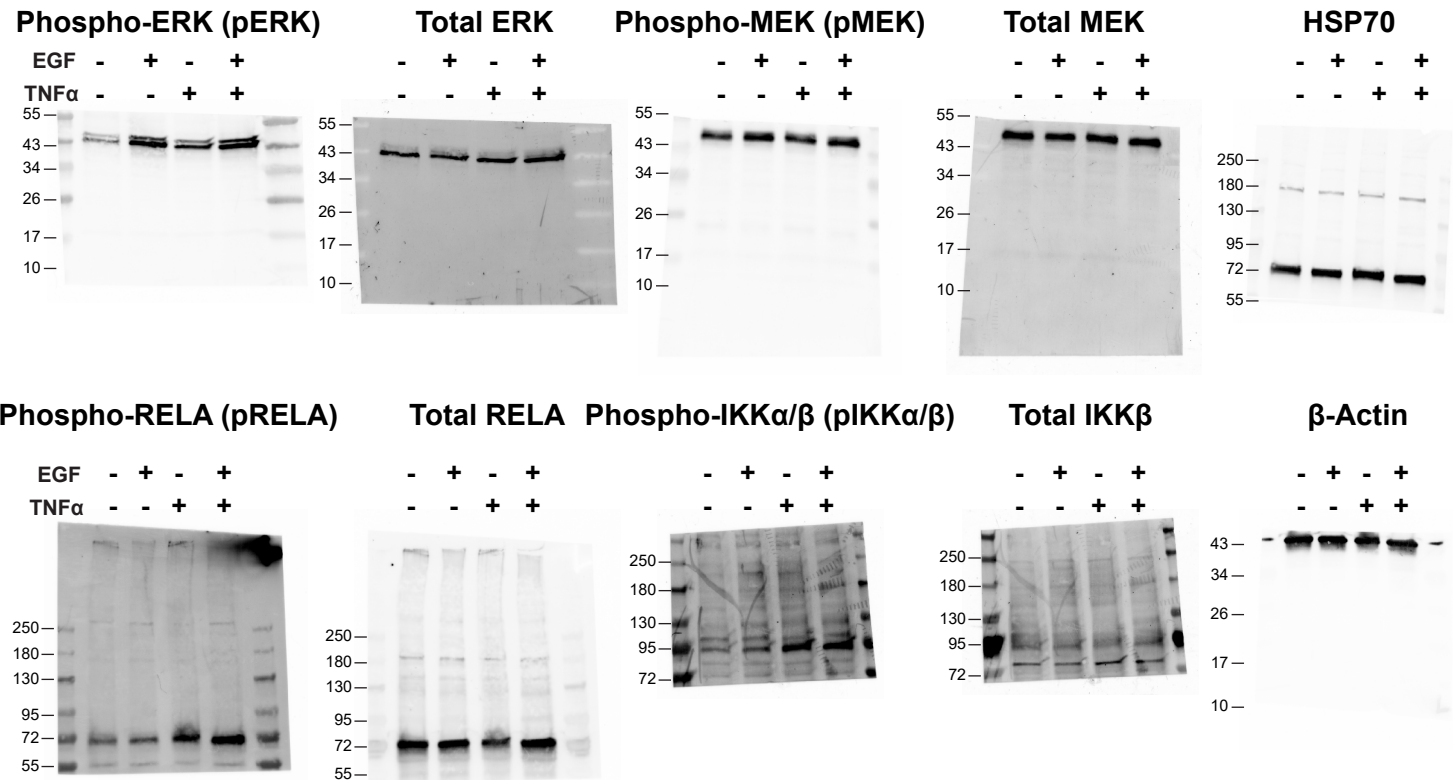

L3.6pl | Figure 3d

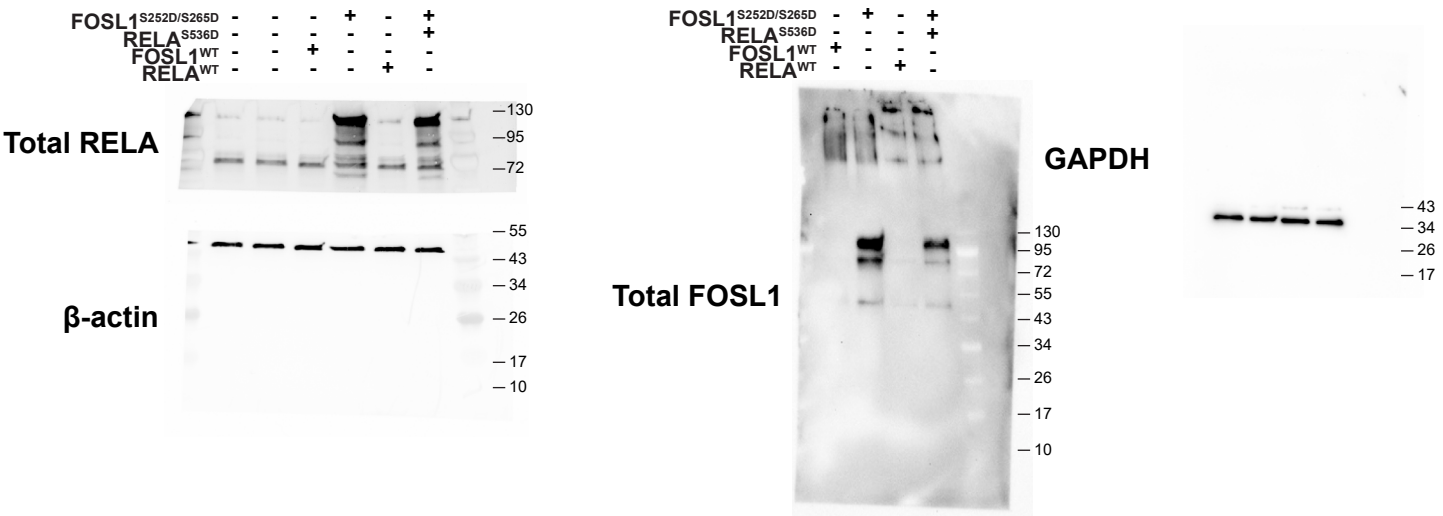

L3.6pl | Figure S4d

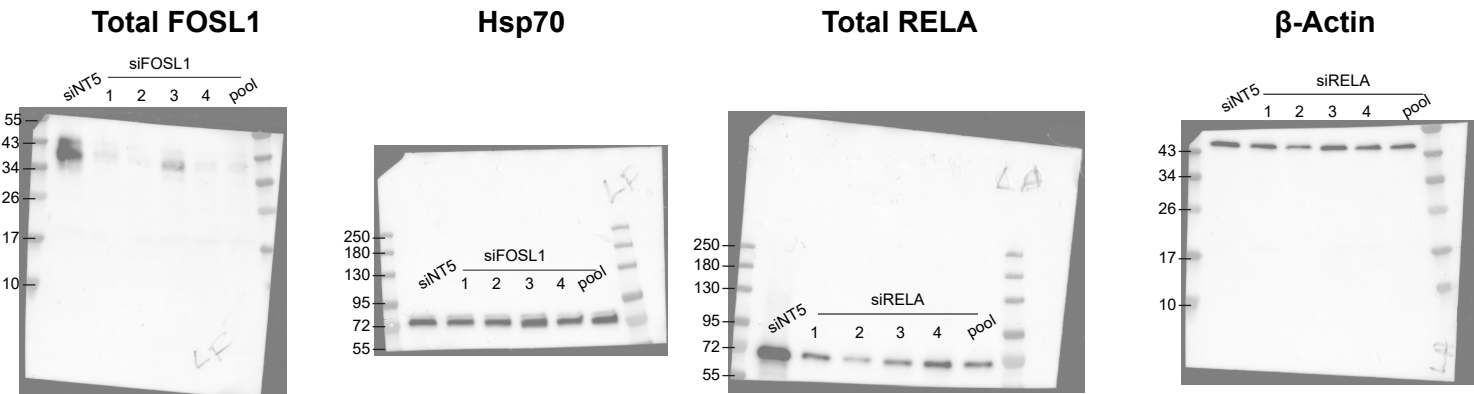

Supplement: Supplementary file 23 — Uncut Western Blot Images [file 41419_2025_7810_MOESM23_ESM.pdf]
