# Supplementary material for: Therapeutic targeting of FOSL1 and RELA-dependent transcriptional mechanisms to suppress pancreatic cancer metastasis
Source: Cell Death Dis. 2025 Jul 9;16(1):504. doi: 10.1038/s41419-025-07810-x (PMC12241458; doi:10.1038/s41419-025-07810-x)
Supplement: Supplementary file 4 — Supplementary Figure S3 [file 41419_2025_7810_MOESM4_ESM.pdf]

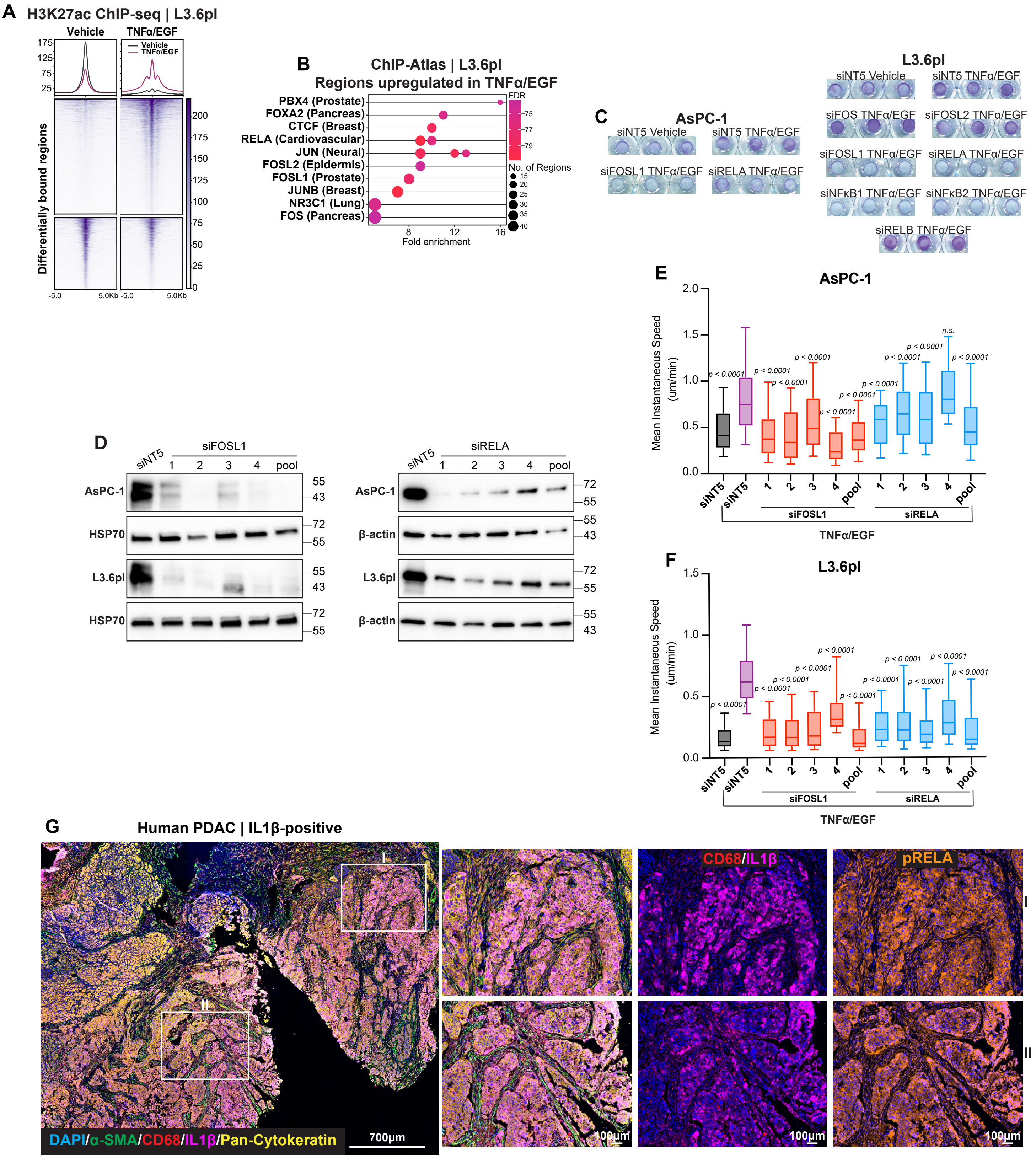

A H3K27ac ChIP-seq | L3.6pl
Vehicle
TNFα/EGF
Differentially bound regions
B ChIP-Atlas | L3.6pl
Regions upregulated in TNFα/EGF
PBX4 (Prostate)
FOXA2 (Pancreas)
CTCF (Breast)
RELA (Cardiovascular)
JUN (Neural)
FOSL2 (Epidermis)
FOSL1 (Prostate)
JUNB (Breast)
NR3C1 (Lung)
FOS (Pancreas)
Fold enrichment
FDR
No. of Regions
C AsPC-1
siNT5 Vehicle
siNT5 TNFα/EGF
siFOSL1 TNFα/EGF
siRELA TNFα/EGF
L3.6pl
siFOS TNFα/EGF
siFOSL2 TNFα/EGF
siNFκB1 TNFα/EGF
siNFκB2 TNFα/EGF
siRELB TNFα/EGF
D siFOSL1
siRELA
siNT5
1 2 3 4 pool
AsPC-1
HSP70
L3.6pl
β-actin
E AsPC-1
Mean Instantaneous Speed (um/min)
p < 0.0001
n.s.
siFOSL1
siRELA
TNFα/EGF
F L3.6pl
G Human PDAC | IL1β-positive
DAPI/α-SMA/CD68/IL1β/Pan-Cytokeratin
700µm
CD68/IL1β
pRELA
I
II
100µm
