# Supplementary material for: Therapeutic targeting of FOSL1 and RELA-dependent transcriptional mechanisms to suppress pancreatic cancer metastasis
Source: Cell Death Dis. 2025 Jul 9;16(1):504. doi: 10.1038/s41419-025-07810-x (PMC12241458; doi:10.1038/s41419-025-07810-x)
Supplement: Supplementary file 5 — Supplementary Figure S4 [file 41419_2025_7810_MOESM5_ESM.pdf]

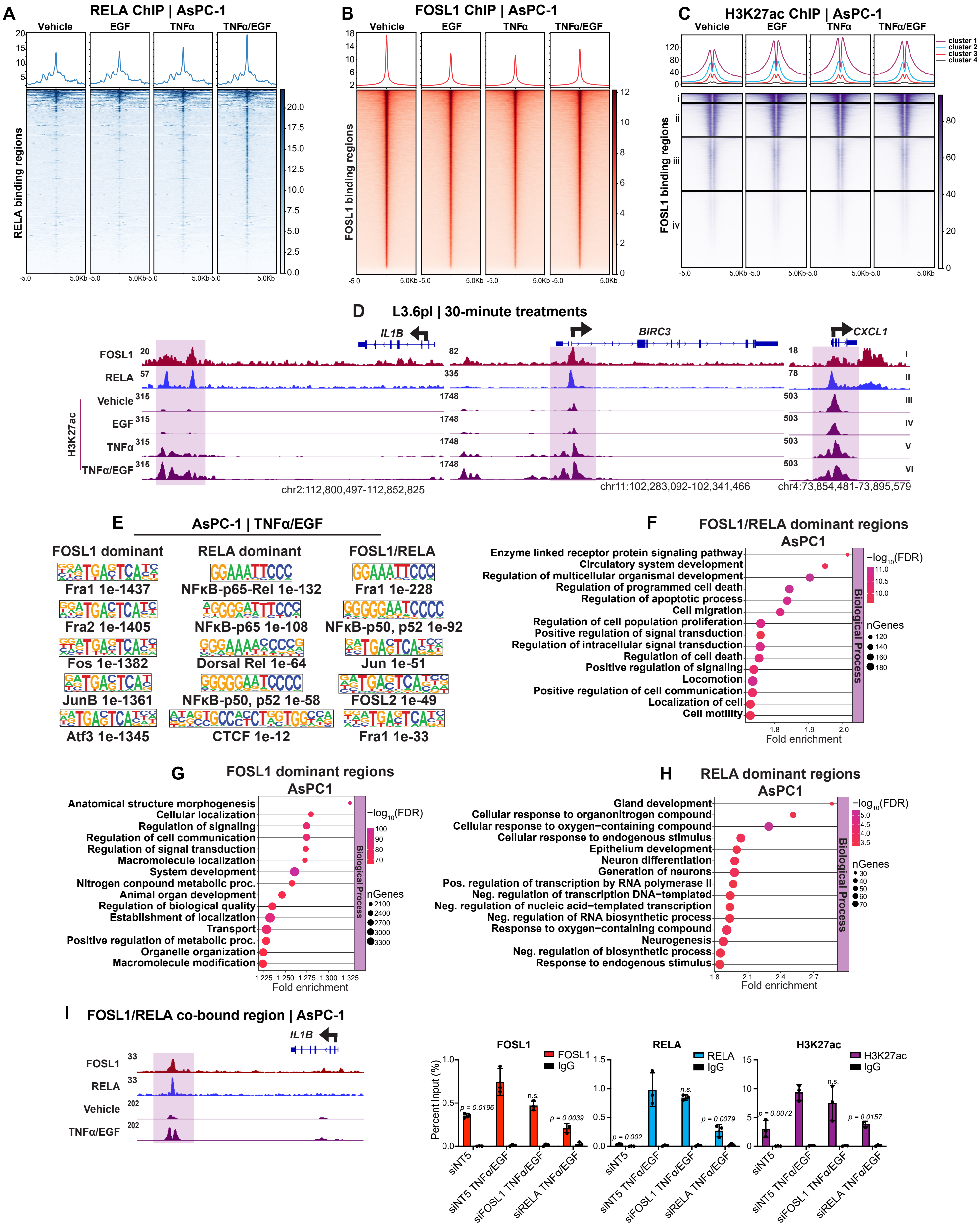
A
RELA ChIP | AsPC-1
Vehicle
EGF
TNFα
TNFα/EGF
RELA binding regions
B
FOSL1 ChIP | AsPC-1
FOSL1 binding regions
C
H3K27ac ChIP | AsPC-1
cluster 1
cluster 2
cluster 3
cluster 4
i
ii
iii
iv
D
L3.6pl | 30-minute treatments
IL1B
BIRC3
CXCL1
FOSL1
RELA
H3K27ac
chr2:112,800,497-112,852,825
chr11:102,283,092-102,341,466
chr4:73,854,481-73,895,579
E
AsPC-1 | TNFα/EGF
FOSL1 dominant
RELA dominant
FOSL1/RELA
Fra1 1e-1437
NFκB-p65-Rel 1e-132
Fra1 1e-228
Fra2 1e-1405
NFκB-p65 1e-108
NFκB-p50, p52 1e-92
Fos 1e-1382
Dorsal Rel 1e-64
Jun 1e-51
JunB 1e-1361
NFκB-p50, p52 1e-58
FOSL2 1e-49
Atf3 1e-1345
CTCF 1e-12
Fra1 1e-33
F
FOSL1/RELA dominant regions
AsPC1
Enzyme linked receptor protein signaling pathway
Circulatory system development
Regulation of multicellular organismal development
Regulation of programmed cell death
Regulation of apoptotic process
Cell migration
Regulation of cell population proliferation
Positive regulation of signal transduction
Regulation of intracellular signal transduction
Regulation of cell death
Positive regulation of signaling
Locomotion
Positive regulation of cell communication
Localization of cell
Cell motility
Biological Process
Fold enrichment
−log10(FDR)
nGenes
G
FOSL1 dominant regions
AsPC1
Anatomical structure morphogenesis
Cellular localization
Regulation of signaling
Regulation of cell communication
Regulation of signal transduction
Macromolecule localization
System development
Nitrogen conpound metabolic proc.
Animal organ development
Regulation of biological quality
Establishment of localization
Transport
Positive regulation of metabolic proc.
Organelle organization
Macromolecule modification
Biological Process
Fold enrichment
H
RELA dominant regions
AsPC1
Gland development
Cellular response to organonitrogen compound
Cellular response to oxygen−containing compound
Cellular response to endogenous stimulus
Epithelium development
Neuron differentiation
Generation of neurons
Pos. regulation of transcription by RNA polymerase II
Neg. regulation of transcription DNA−templated
Neg. regulation of nucleic acid−templated transcription
Neg. regulation of RNA biosynthetic process
Response to oxygen−containing compound
Neurogenesis
Neg. regulation of biosynthetic process
Response to endogenous stimulus
Biological Process
Fold enrichment
I
FOSL1/RELA co-bound region | AsPC-1
IL1B
FOSL1
RELA
Vehicle
TNFα/EGF
FOSL1
RELA
H3K27ac
IgG
Percent Input (%)
p = 0.0196
n.s.
p = 0.0039
p = 0.002
p = 0.0079
p = 0.0072
p = 0.0157
siNT5
siNT5 TNFα/EGF
siFOSL1 TNFα/EGF
siRELA TNFα/EGF
