# Supplementary material for: Therapeutic targeting of FOSL1 and RELA-dependent transcriptional mechanisms to suppress pancreatic cancer metastasis
Source: Cell Death Dis. 2025 Jul 9;16(1):504. doi: 10.1038/s41419-025-07810-x (PMC12241458; doi:10.1038/s41419-025-07810-x)
Supplement: Supplementary file 7 — Supplementary Figure S6 [file 41419_2025_7810_MOESM7_ESM.pdf]

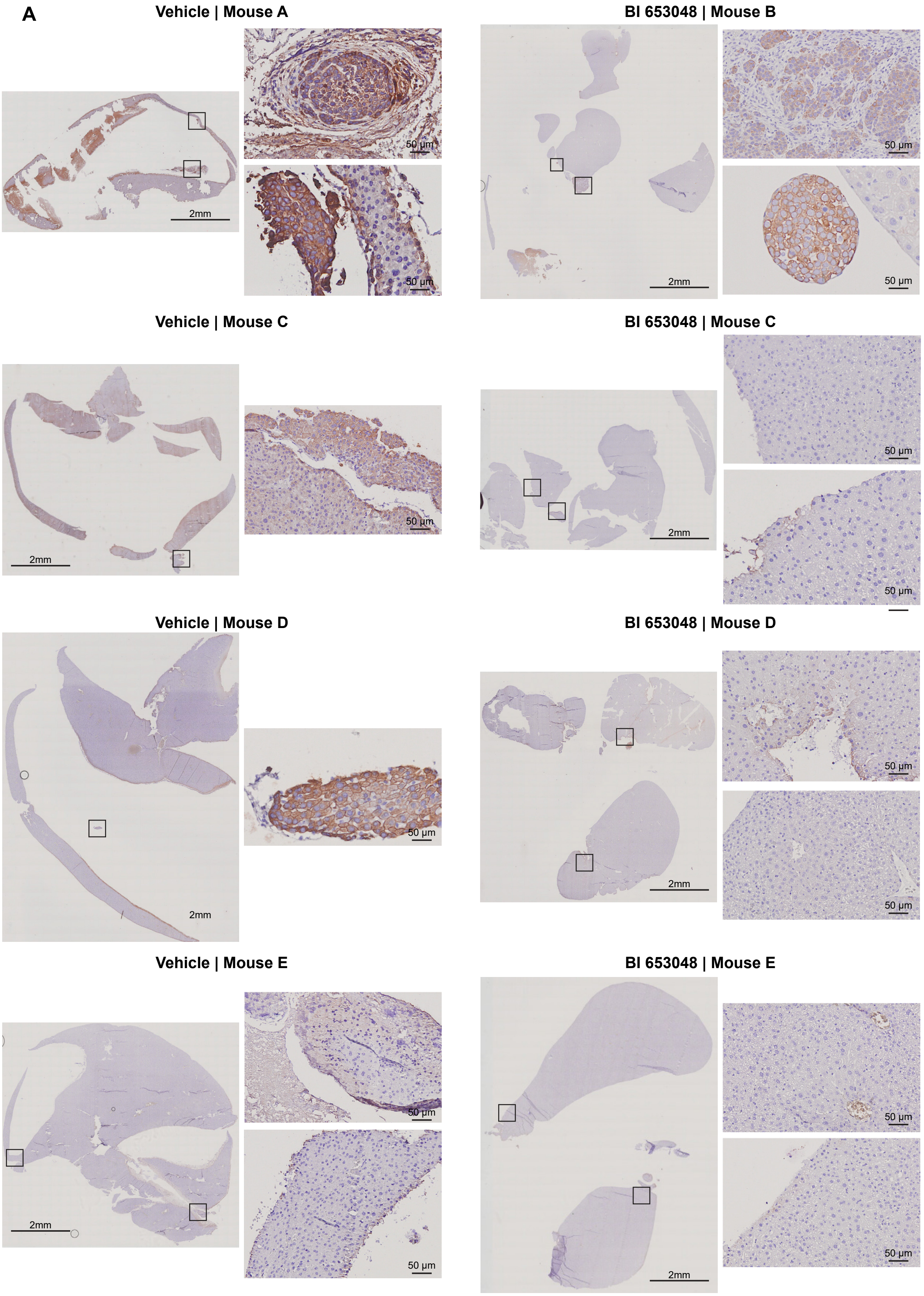

A
Vehicle | Mouse A
BI 653048 | Mouse B
Vehicle | Mouse C
BI 653048 | Mouse C
Vehicle | Mouse D
BI 653048 | Mouse D
Vehicle | Mouse E
BI 653048 | Mouse E
2mm
50 µm
