# Supplementary material for: Therapeutic targeting of FOSL1 and RELA-dependent transcriptional mechanisms to suppress pancreatic cancer metastasis
Source: Cell Death Dis. 2025 Jul 9;16(1):504. doi: 10.1038/s41419-025-07810-x (PMC12241458; doi:10.1038/s41419-025-07810-x)

Supplementary material 1: western blots used in this study

AsPC-1 | Figure 2b

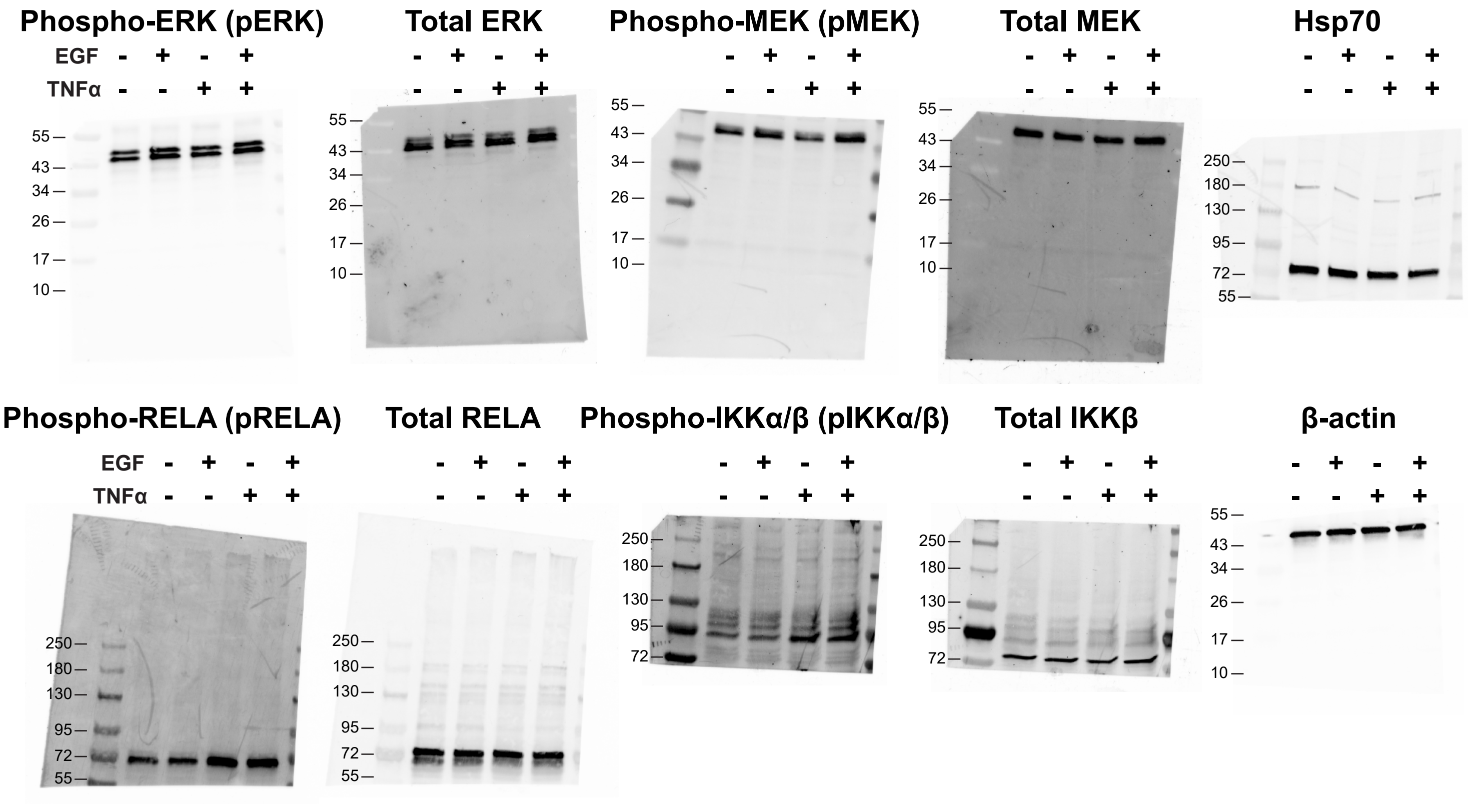

AsPC-1 | Figure 3d

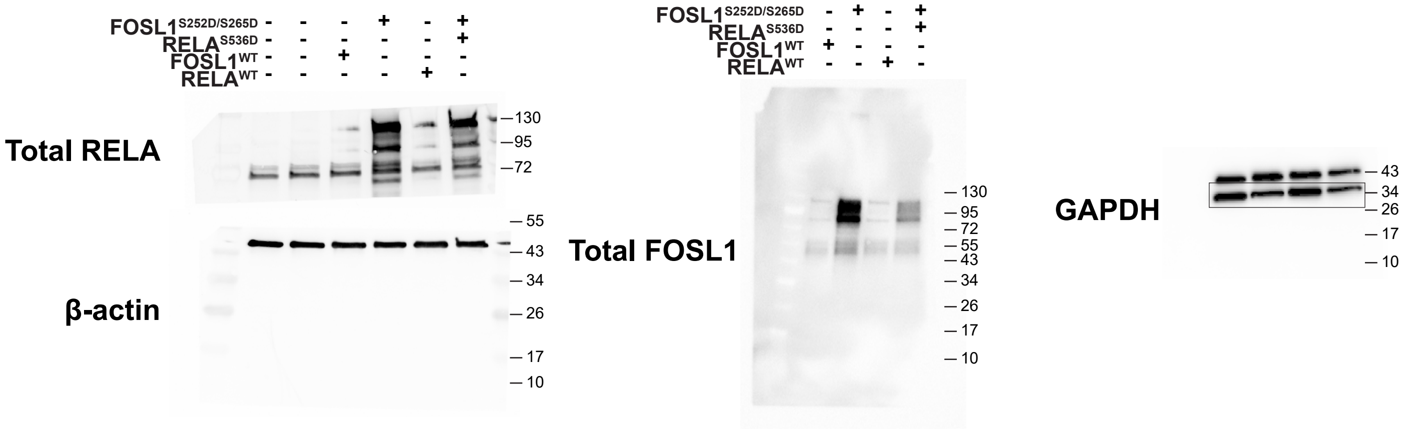

AsPC-1 | Figure S4d

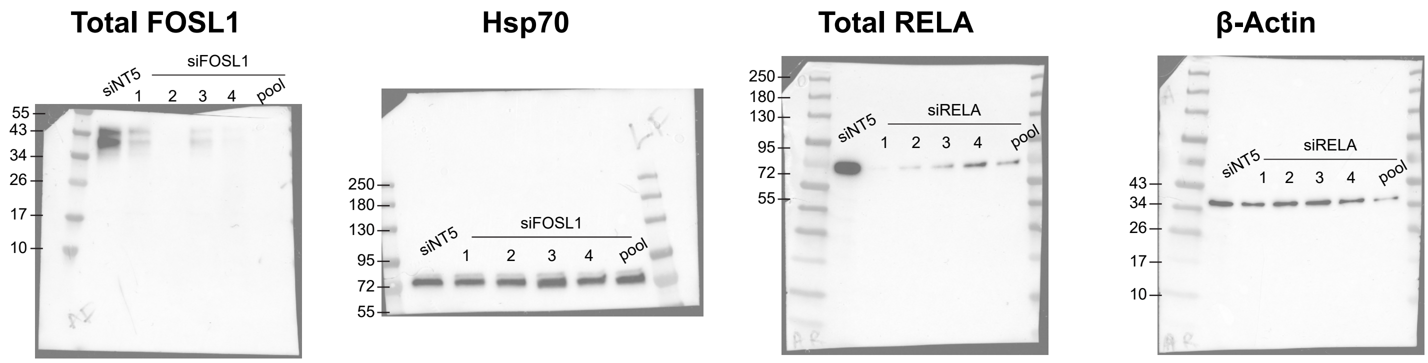

Supplement: Supplementary file 22 — Uncut Western Blot Images [file 41419_2025_7810_MOESM22_ESM.pdf]
